# Scaffolding of long read assemblies using long range contact information

**DOI:** 10.1186/s12864-017-3879-z

**Published:** 2017-07-12

**Authors:** Jay Ghurye, Mihai Pop, Sergey Koren, Derek Bickhart, Chen-Shan Chin

**Affiliations:** 1 Department of Computer Science, University of Maryland, 20742 College Park, Maryland, USA; 2 Genome Informatics Section, Computational and Statistical Genomics Branch, National Human Genome Research Institute, National Institutes of Health, 21702 Bethesda, Maryland, USA; 3 Pacific Biosciences, 94205 Menlo Park, California, USA; 4 Cell Wall Biology and Utilization Research, US Dairy Forage Research Center, 53706 Madison, Wisconsin, USA

**Keywords:** Assembly, Scaffolding, Hi-C, Long reads

## Abstract

**Background:**

Long read technologies have revolutionized de novo genome assembly by generating contigs orders of magnitude longer than that of short read assemblies. Although assembly contiguity has increased, it usually does not reconstruct a full chromosome or an arm of the chromosome, resulting in an unfinished chromosome level assembly. To increase the contiguity of the assembly to the chromosome level, different strategies are used which exploit long range contact information between chromosomes in the genome.

**Methods:**

We develop a scalable and computationally efficient scaffolding method that can boost the assembly contiguity to a large extent using genome-wide chromatin interaction data such as Hi-C.

**Results:**

we demonstrate an algorithm that uses Hi-C data for longer-range scaffolding of de novo long read genome assemblies. We tested our methods on the human and goat genome assemblies. We compare our scaffolds with the scaffolds generated by LACHESIS based on various metrics.

**Conclusion:**

Our new algorithm SALSA produces more accurate scaffolds compared to the existing state of the art method LACHESIS.

**Electronic supplementary material:**

The online version of this article (doi:10.1186/s12864-017-3879-z) contains supplementary material, which is available to authorized users.

## Background

The advent of massively parallel sequencing technologies has made the generation of billions of reads possible at a very low-cost per sequenced base. Despite the progress made in *de novo* assembly algorithms, quality of short read assemblies is far from what is necessary for effective further analysis due to the fundamental limit - the read length is shorter than repeat lengths for the majority of repeat classes [1, 2]. For example, short read *de novo* assemblies of the human genome are highly fragmented compared to the chromosomes of the *H.sapiens* reference [3, 4]. Thus, high throughput sequencing technology has reached a point where increasing sequencing coverage of short reads does not significantly improve assembly quality. Recent advances in single-molecule sequencing technologies have provided reads almost 100 times longer than second generation methods [5]. Most prominently, Pacific Biosciences’ single molecule real time (SMRT) sequencing delivers reads of lengths up to 50 Kbp [6] whereas Oxford Nanopore’s nanopore sequencing can deliver read lengths greater than 10 Kbp [7]. Since these reads are likely to be longer than most common repeats, they drastically reduce the complexity caused by repeats during the assembly process. However, such long reads suffer from low accuracy which requires new algorithms for their assembly. It has been shown that SMRT long reads follow a random error model [8, 9], due to which near perfect assembly is possible despite the high error rate [10]. Hence by sampling the genome at sufficient coverage, SMRT sequencing has been used to produce assemblies of unprecedented contiguity [11–14]. Although long read technologies have made the resolution of highly repetitive regions possible, the contigs generated from long read assembly do not always span a complete chromosome or even an arm of the chromosome. To get chromosome scale scaffolds, various strategies have been explored to increase the contiguity of *de novo* genome assemblies. Some of these strategies rely on end sequencing of fosmid clones [3], fosmid clone dilution pool sequencing [15], optical mapping [16–19], linked-read sequencing [20, 21] and synthetic long reads [22–24]. The central principle of all these strategies is to find linkage information connecting distant regions of the chromosome and use that information to orient and order contigs with respect to each other. Some of the newer technologies like Hi-C use proximity ligation and massively parallel sequencing to probe the three-dimensional structure of chromosomes within the nucleus and capture interactions by paired-end sequencing [25, 26]. In the data generated by the Hi-C protocol, the intrachromosomal contact probability is on average much higher than the interchromosomal contact probability [27, 28]. Regions separated by several hundred megabases on the same chromosome are more likely to interact than regions on different chromosomes, though it is important to note that the interaction probability rapidly decays with increasing genomic distance [25]. The main advantage of Hi-C over previous methods is the ability to capture interactions over much larger genomic distances thereby producing scaffolds which can span a complete arm of the chromosome.

Several efforts have been made to use Hi-C data to scaffold the ‘draft stage’ short read assemblies. Burton et al. [27] developed a computational approach in their tool LACHESIS that combined Hi-C data with short read assemblies to generate chromosome level scaffolds. They used their methods to scaffold *de novo* assemblies of human, mouse and fruitfly. LACHESIS uses the Hi-C reads alignments to contigs to cluster contigs into one cluster per chromosome with hierarchical clustering. To order the contigs in each cluster, it first finds the maximum spanning tree for the graph corresponding to each cluster. It then finds the longest path in the spanning tree which represents the initial contig ordering. After this, it reinserts contigs which are not part of the initial ordering into the longest path yielding the final contig ordering for each cluster. Once the ordering is computed, it constructs a weighted directed acyclic graph (WDAG) encoding all possible ways in which contigs can be oriented, with score assigned to each orientation. Finally, it finds the heaviest path through this WDAG describing the optimal orientation assigned to the ordered contigs in each cluster. The primary drawback of LACHESIS is that it needs the number of clusters to be pre-specified. This method can not be applied to scaffold the contigs of genomes when the number of chromosomes in the organisms are unknown. Kaplan et al. [28] developed a method for scaffolding based on statistical techniques. Their method uses the hierarchical clustering method similar to LACHESIS, but it predicts the number of clusters. The major drawback of their method is that they do not orient the contigs in each cluster, thereby not providing complete information needed for scaffolding. Also, most of the experiments performed using their method used simulated contigs of equal size, except for the scaffolding of chromosome 14. Due to this, it is unclear how their method would perform in the case of long read assemblies where contig lengths can have large variance. Since both of these methods rely on hierarchical clustering, it is expensive to compute all vs all link scores for all the contigs, causing scalability issues. Another drawback of both methods is that they do not detect and correct misassemblies before scaffolding. If assembly errors are not corrected, it could result in erroneous scaffolds and may also propagate errors across multiple scaffolds causing misjoins. Marie-Nelly et al. [29] developed a probabilistic method called GRAAL to generate scaffolds from Hi-C data. However, their validation was limited to a single chromosome (Chr 14) of the human genome and we were unable to run it on full vertebrate genomes. Hence, it is unclear how their method performs in terms of runtime and accuracy for the scaffolding of all chromosomes of the human genome.

In our work, we address the issues in the previous methods. We make use of genome-wide chromatin interaction data sets generated by the Hi-C protocol to linearly orient and order assembled contigs along entire chromosomes. We develop a scaffolding tool SALSA (**S**imple **A**ssemb**L**y **S**c**A**ffolder) based on a computational method that exploits the genomic proximity information in Hi-C data sets for long range scaffolding of *de novo* genome assemblies. We tested SALSA on its ability to reconstruct the human and goat genome. Our method can produce centromere to telomere scaffolds of chromosomes in most cases and telomere to telomere scaffolds in best cases.

## Results and discussion

### Contact probability of hi-C data

**Fig. 1:**
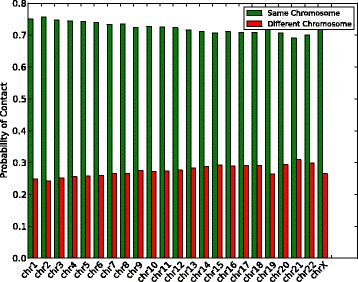
The probability of contact calculated based on read mapping to GRCh38 reference

We aligned Hi-C reads from NA12878, a human genome used in the 1000 Genomes project [30, 31], to the GRCh38 human reference genome [32] using BWA mem (version - 0.7.13) [33] with default parameters. If both mates in the read pair align to the same chromosome, it implies an intrachromosomal contact. For each chromosome, we count how many read pairs have both mates mapped to that chromosome and how many reads have just one of the mates mapped to that chromosome. Using this information, we compute the intrachromosomal and interchromosomal contact probability for each chromosome. It can be seen from Fig. 1 that the probability of intrachromosomal contact is much higher than that of interchromosomal contact.

### Scaffolding assemblies of two genomes

We tested the effectiveness of our approach, at the chromosome scale, on the de novo assembly of two genomes. We used two assemblies, one was NA 12,878 with scaffold NG50 of 26.83 Mb [30, 31] and the other was the genome of *Capra hircus* (goat) with contig NG50 of 3.86 Mb [34]. After aligning Hi-C reads to both assemblies, we used our algorithm to construct the scaffold graph and later to orient and order the contigs. For the NA12878 assembly, it generated 1555 scaffolds with NG50 of 60.02 Mb. For the *Capra hircus* assembly, it generated 127 scaffolds with NG50 of 58.64 Mb. Table 1 summarizes the statistics of the contig and scaffold assemblies.

**Table 1:** Contig and Scaffold statistics for NA 12878 and *Capra hircus*

Metric	NA12878	*Capra hircus*
Number of contigs	18903	33767
NG50	26.83 Mb	3.86 Mb
Number of scaffolds	1555	127
Scaffold NG50	60.02 Mb	58.64 Mb
Total bases	2.77 Gb	2.94 Gb

### Comparison of SALSA with LACHESIS

To assess the quality of the scaffolds, we aligned them to their respective reference genomes using *nucmer* program (parameters : -c 1000 -l 100) in MUMmer package [35]. To evaluate the accuracy of NA12878 scaffolds, we used the (GRCh38) human reference genome. To evaluate accuracy of the goat scaffolds, we used the recently published goat reference genome (BioProject PRJNA290100) [34]. The alignment quality was assessed using *dnadiff* [36], a program which evaluates draft assemblies based on a set of metrics by aligning the scaffolds to the reference genome. We focused on four metrics: 1) The number of breakpoints, defined as the number of gap between pairs of mutually consistent alignments that did not occur due to the end of a sequences, which also include gaps (stretches of N) in the assembly scaffolds. 2) The number of relocations, defined as the number of breaks in the alignment caused by consecutively aligned sequences belonging to the same chromosome in an incorrect order. This accounts for the ordering differences in the scaffold construction. 3) The number of translocations, defined as the count of the number of breaks in the alignment where consecutively aligned sequences belong to different chromosomes. This accounts for the inter-chromosomal join errors in the scaffold. 4) The number of inversions, defined as the number of breaks in the alignment caused when a contig in the scaffold is inverted relative to it’s orientation implied by the reference genome.

Table 2 shows the comparison of scaffolds generated by SALSA and LACHESIS for NA12878 assembly. The number of relocations is higher in LACHESIS implying that LACHESIS produced many differences while ordering contigs belonging to a particular chromosome [34]. Also, LACHESIS scaffolds contain 66 more inversions compared to SALSA scaffolds. The number of breakpoints is higher in SALSA scaffolds compared to LACHESIS scaffolds. Since the NA12878 genome likely has true structural differences from the GRCh38 reference [31, 37], some of the differences are shared by both the assemblies. These are more likely to be true variations. In contrast, differences present in only one of the assemblies are more likely errors introduced by the scaffolder. We identified 10,526 breakpoints common to both scaffolders, indicating that a third of breakpoints are likely true variations. To differentiate the scaffolding errors from the structural variants, we found out the errors unique to SALSA and LACHESIS scaffolds. If an error is not present in both the scaffolds, then it is more likely an artifact of errors introduced by the scaffolding method. We found that SALSA had 94 relocations, 282 inversions and 64 inter-chromosomal joins unique to it whereas LACHESIS had 331 relocations, 348 inversions and 47 inter-chromosomal joins unique to it. Therefore, considering just differences unique to a scaffolder, LACHESIS still has a higher count that SALSA.

**Table 2:** Evaluation of scaffolds generated by SALSA and LACHESIS for the human NA12878 assembly

Metric	SALSA	LACHESIS
Number of scaffolds	1555	23
Total bases	2.92 Gb	2.79 Gb
NG50	60.02 Mb	143.802 Mb
% Aligned bases	94.52%	94.72%
Breakpoints	33079	26288
Relocations	136	373
Translocations	96	79
Inversions	408	474

**Fig. 2:**
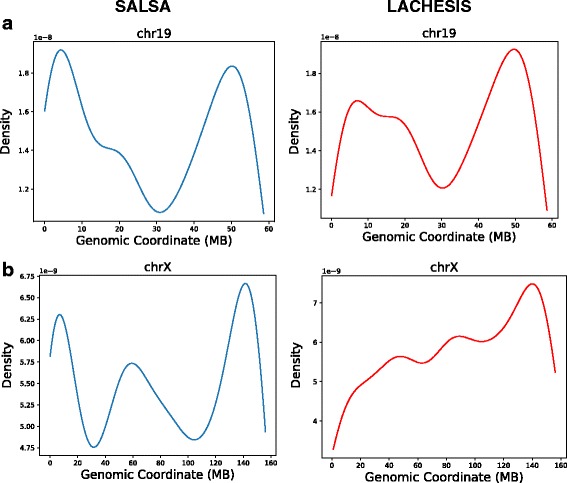
Density plot for misassemblies as a function of the position on the chromosome. **a** For chromosome 19, most of the errors generated by SALSA are concentrated near telomeres, whereas the errors generated by lachesis are uniformly spread across the p-arm. **b** For Chromosome X, the errors generated by SALSA are heavily concentrated near centromere and telomeres, but the errors generated by LACHESIS are spread uniformly across the entire chromosome X with a peak near telomere

To further understand the locations on the chromosome where both SALSA and LACHESIS have misassemblies, we generated a density plot of number of differences compared to the reference as a function of the location on the chromosome. Regions such as centromeres and telomeres of the human chromosomes are repetitive and difficult to resolve unambiguously, thus generating more differences compared to other regions. Figure 2 shows the density plot for chromosome 19 and chromosome X. It can be observed that for chromosome 19, most of the differences generated by SALSA are concentrated near telomeres, whereas differences generated by LACHESIS are uniformly spread across the p-arm of chromosome 19. For chromosome X, the differences generated by SALSA are concentrated near centromere and telomeres and have very low density in the remaining regions. However, the differences generated by LACHESIS are evenly spread across the entire chromosome with a slight peak near the telomere of the q-arm. Thus, it shows that SALSA generates better scaffolds than LACHESIS outside of low-complexity regions (centromeres and telomeres).

Table 3 shows the comparison of scaffolds generated by SALSA and LACHESIS for the *Capra hircus* assembly. While both SALSA and LACHESIS scaffolds have a similar proportion of aligned bases to reference, LACHESIS produced many more inversions and relocations compared to SALSA. SALSA produced 105 inversions and 67 relocations compared to 439 inversions and 374 relocations produced by LACHESIS. SALSA also produced fewer inter-chromosomal mis-join errors (213) compared to LACHESIS(601). To normalize the errors for the assembly size difference between LACHESIS and SALSA, we also computed the errors made by LACHESIS scaffolds only in the regions covered by SALSA scaffolds. In these regions, LACHESIS made 432 inversions, 363 relocations and 592 inter-chromosomal join errors. The majority of differences generated by LACHESIS occur in the genomic region covered by both LACHESIS and SALSA.

**Table 3:** Evaluation of scaffolds generated by SALSA and LACHESIS for the *Capra hircus* assembly

Metric	SALSA	LACHESIS
Number of scaffolds	127	990
Total bases	2.44 Gb	2.62 Gb
NG50	46.64 Mb	88.79 Mb
% Aligned bases	99.88%	99.85%
Breakpoints	8514	14035
Relocations	67	374
Translocations	213	601
Inversions	105	439

**Fig. 3:**
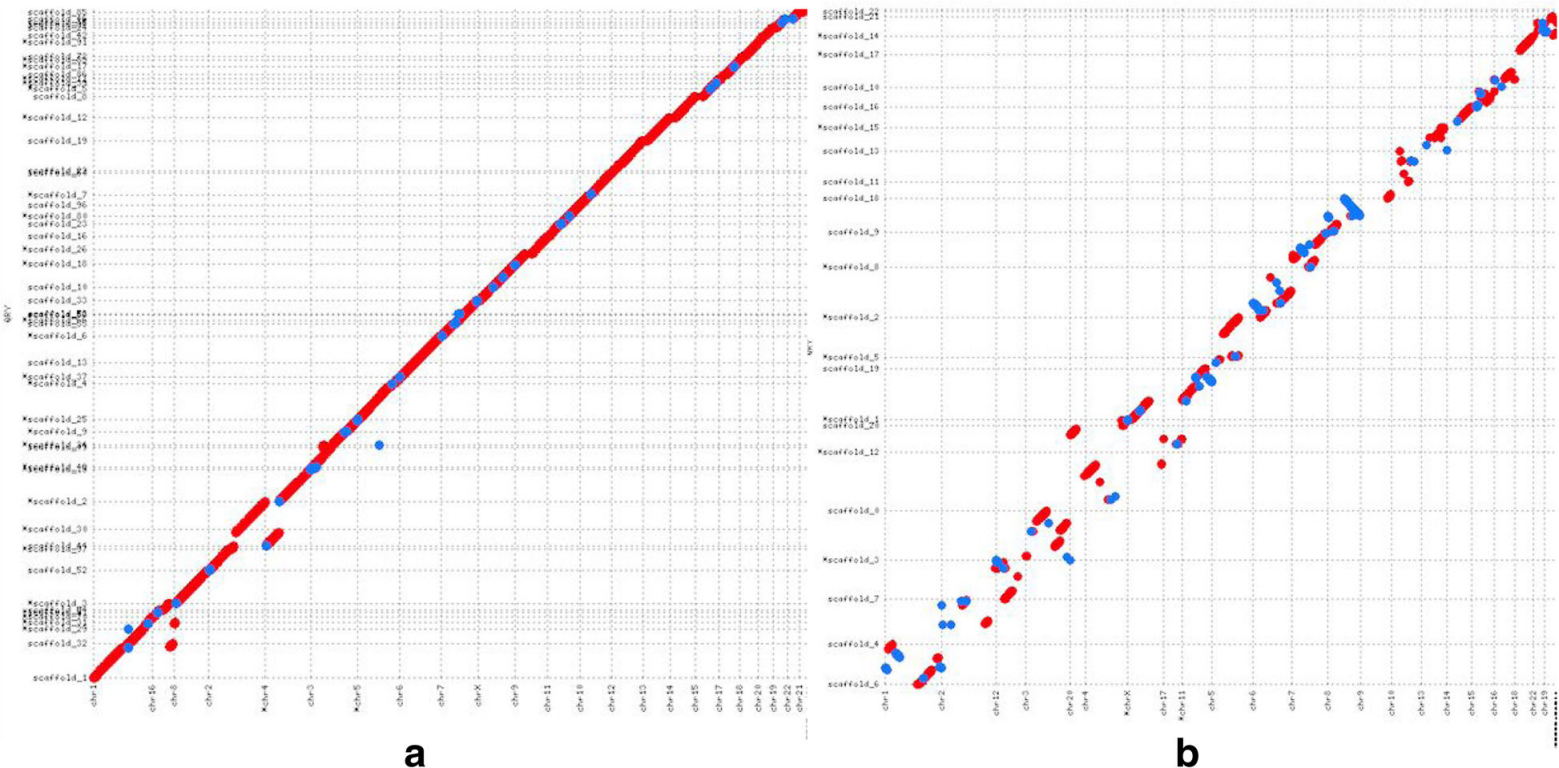
**a** The alignment dotplot for NA12878 scaffolds generated by SALSA. **b** The alignment dotplot of Lachesis scaffolds for NA12878

**Fig. 4:**
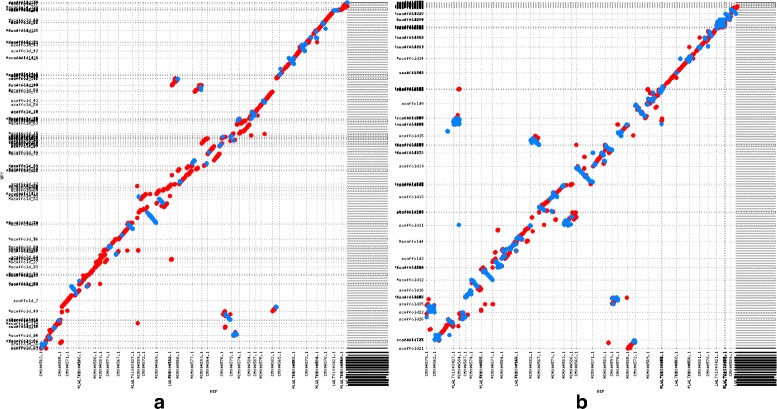
**a** The alignment dotplot for *Capra hircus* scaffolds generated by SALSA. **b** The alignment dotplot of Lachesis scaffolds for *Capra hircus*

Figures 3 and 4 show the alignment dotplots for the human and goat scaffolds respectively when aligned to their respective reference genomes. In the case of NA12878 scaffolds, it is observed that (Fig. 3 a) the scaffolds generated by LACHESIS are highly fragmented. On the other hand, the scaffolds generated by SALSA (Fig. 3 b) are more contiguous, contain fewer inversions and relocations and are more consistent with the reference than the LACHESIS scaffolds. Dot plots, displaying the alignments for each chromosome can be found in the (Additional file 1: Figure S1). In the case of the goat scaffolds, although LACHESIS produced more contiguous scaffolds, it incurred many large scale inversions and relocations (Fig. 4 a). In contrast, SALSA is able to produce contiguous scaffolds with fewer orientation and ordering errors compared to LACHESIS thereby producing scaffolds that are highly consistent with the reference.

### Scaffolding optical map scaffolds

We used the goat genome assembly scaffolded with optical map data to test the accuracy of SALSA [34]. The initial scaffold assembly had 1575 scaffolds with NG50 of 20.62 Mb which is much more contiguous than the long read assembly. After scaffolding with the optical maps, SALSA produced 90 scaffolds whereas LACHESIS produced 596 scaffolds. We evaluated these scaffolds using the metrics described before and the results are shown in Table 4. It is observed that even though the NG50 of the input assembly is high, LACHESIS is still prone to relocations compared to SALSA. However, in this case, SALSA produced 3.17% more inversions than LACHESIS. There were fewer differences produced by LACHESIS in this case for a couple of reasons. First, the misassemblies in the original assembly were corrected with the optical map data. This helped LACHESIS, since it does not have a built-in misassembly detection method like SALSA. Second, the scaffold assembly with optical maps was highly contiguous compared to the original assembly (20.62 Mb vs 3.86 Mb NG50, respectively) which improved the scaffold quality generated by LACHESIS. Using the uncorrected input contigs, SALSA’s assembly had fewer differences than LACHESIS and did so without the need for further error correction by another scaffolding technology. The dotplots for both assembly’s scaffolds aligned to reference genomes are shown in the (Additional file 1: Figure S2).

**Table 4:** Evaluation of scaffolds generated by SALSA and LACHESIS for *Capra hircus* assembly generated using optical map data

Metric	SALSA	LACHESIS
Number of scaffolds	90	596
Total bases	2.22 Gb	2.61 Gb
NG50	87.74 Mb	87.34 Mb
% Aligned bases	97.00%	99.91%
Breakpoints	10718	14741
Relocations	118	167
Translocations	185	407
Inversions	130	126

### Validation using RH mapping

Radiation hybrid (RH) mapping [38] uses X-ray breakage of DNA to determine distance and order between DNA markers on the chromosome. This validation is useful because it spots errors in the scaffolds without the reference genome. We used the RH map for the goat genome generated by Du et al. [26] to validate scaffolds generated with SALSA and compared it with LACHESIS. In particular, we focused on three types of errors. First is orientation errors, which occur when the orientation of contigs in a particular scaffold is different than the RH map implied orientation. Second is the incorrect chromosome error, which occurs when for a particular scaffold, a contig in that scaffold is assigned to a different chromosome than the chromosome assigned to the majority of the contigs by RH map. The third is ordering errors, which occur when the ordering of contigs in a scaffold is not the same as the ordering implied by RH map.

Table 5 shows the errors produced by SALSA and LACHESIS scaffolds generated from the long read assembly and optical map scaffolds when validated with RH map. In the scaffolds from the long read assembly, SALSA produced 146 orientation errors compared to 600 orientation errors produced by LACHESIS. SALSA also produced far fewer ordering errors compared to LACHESIS. However, SALSA produced more incorrect chromosome errors(52) compared to LACHESIS(32). When optical map scaffolds were used, SALSA produces 63 ordering and 33 orientation errors whereas LACHESIS produced 78 ordering and 31 orientation errors. In this case, LACHESIS assigned correct chromosome to all the scaffolds, whereas SALSA failed to assign a correct chromosome to 21 scaffolds. It can be seen that, for both the scaffolds, SALSA produces far fewer orientation and ordering errors compared to LACHESIS, but LACHESIS is better at assigning correct chromosomes to the scaffolds. This is because, when the number of clusters is specified for LACHESIS, it has an advantage on the accuracy of the contig to chromosome assignment. However, for most of the newly sequenced genomes, the correct number of chromosomes is not known in a prior. In such case, if LACHESIS is run with an approximate number of chromosomes as an input, it can generate clustering errors which can further produce orientation and ordering errors in the final scaffolds [34]. This reference independent validation of scaffolds confirms the effectiveness of our methods.

**Table 5:** RH map evaluation of scaffolds generated by SALSA and LACHESIS for *Capra hircus* assembly

Metric	SALSA	LACHESIS	SALSA-OM	LACHESIS-OM
Orientation errors	146	600	33	31
Incorrect chromosome errors	52	32	21	0
Ordering errors	152	552	63	78

SALSA-OM and LACHESIS-OM stands for the scaffolds generated on optical map scaffolds by SALSA and LACHESIS respectively

## Conclusion

In this work, we used genome-wide interaction data sets like Hi-C to orient and order contigs into scaffolds and compare with previous method. Since long read assemblies were used, most of the issues cause by repeats were solved. We also use a weight function to normalize the scores of Hi-C links which reduces the length biases inherent in long contigs. Due to the large variance in contig length from long read assemblies, edge weight normalization plays an important role in generating correct scaffolds. We also provide a method to correct mistakes in the assemblies so that these errors do not propagate through the scaffolding process. In the tests performed on the human and goat genomes, our method showed significant improvements over LACHESIS, the current state of the art tool in this emerging field. The primary benefit of SALSA over LACHESIS is the independence on the number of chromosomes. This is especially useful as the exact number chromosomes may not be available or the chromosme size distribution may skew the clustering algorithm. However, designing a clustering method that clusters the contigs without knowing the actual number of desired clusters is required to estimate the number of chromosomes. Most of the orientation and ordering errors in our method occured in the repetitive regions near centromeres and telomeres. One potential solution to overcome this problem is to do pairwise alignment of all the contigs and trim the contigs to mask these repetitive regions [39]. However, such an approach incurs higher computational cost. Our method requires manual tuning of parameters to achieve optimal results. We plan to incorporate automatic parameter detection at runtime to remove the onus of parameter tuning from the user. Our method can be extended to leverage other chromatin interaction datasets such as Dovetail Chicago libraries [40] and can adapt to their chromosomal contact model. As most of the errors in the scaffolds were mistaken inversions, we are planning to use graph output of genome assemblers [41] in future versions of SALSA to mitigate these types of errors.

## Methods

### Aligning Hi-C Reads

Hi-C reads were aligned to long read assemblies using BWA [33] (version - 0.7.13) with default parameters. Reads with mapping quality ≤ 30, which included the reads mapped more than once were removed from further analysis. Also, only the read pairs with both reads in the pair aligned to contigs are considered for scaffolding.

### Detection of Mis-assemblies

**Fig. 5:**
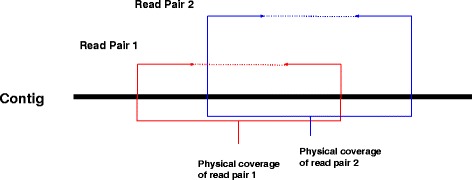
Physical coverage induced by Hi-C read pair. The *solid arrows* denote the read pair and *dotted line* denote the gap between the reads in the pair

Contigs may contain mis-assemblies [36]. We provide a method to detect and correct these misassemblies similar to the one described in [40] using the mapping of Hi-C data to the assembled contigs. For each read pair, its physical coverage is defined as the total bases spanned by the sequence of reads and the gap between the two reads when mapped to contigs (Fig. 5). We also define, per base physical coverage for each base in the contig as the number of read pairs’ physical coverage it is part of. Using these definitions, we compute the physical coverage for each base of all the contigs in the assembly. The misassembly can be detected by the sudden drop in per-base physical coverage in a contig. A particular threshold below which if per base physical coverage falls for contiguous regions in the genome, we call it a misassembly and break contigs at that point. To do this efficiently, we use a variation of Kadane’s algorithm for maximum sum subarray problem [42]. We find the subarray in the array of physical coverage where coverage is consistently low compared to the adjacent regions and use that as the signal for misassembly. (Additional file 1: Figure S3). We performed misassembly detection on the contigs for goat genome assembly. For other datasets, bionano optical maps performs contig error correction and breaking so we did not run our error correction method on those datasets.

### Graph construction and link scoring

**Fig. 6:**
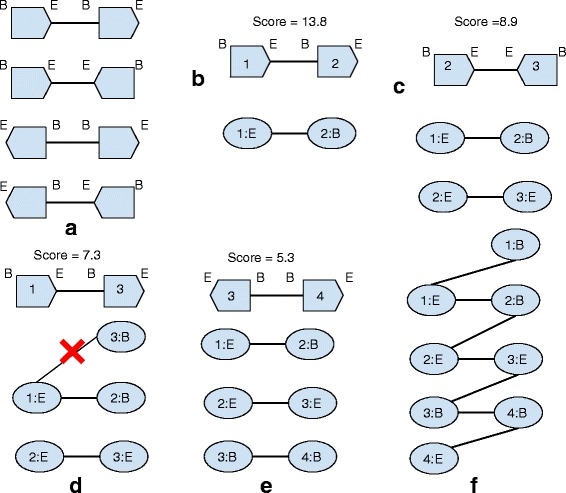
SALSA graph construction method. To construct the graph, links are considered in decreasing order of their score. **a** Types of links present between contigs. The link types can be BB, BE, EB and EE depending on the orientation of the contigs in the link. **b** For each end of the link, a node is created in the graph. In this case 1:E and 2:B nodes are created in the graph with an edge added between them. **c** Similar to **b**, nodes 2:E and 3:E with an edge between them are added to the graph. **d** In this case, node 1:E already has an edge associated to it. So link between 1:E and 3:B is not added to the graph **e** The edge between node 3:B and 4:B is added to the graph since both of them are not present in the graph. **f** After all links are considered, edges are added between B and E nodes of same contigs. This completes the graph construction



We use an idea similar to the string graph formulation in [43] to construct the scaffold graph. The scaffold graph *G*(*V*,*E*,*W*) consists of nodes *V* which represent the end of contigs, edges *E* representing the linkage implied by Hi-C read pairs between ends of two contigs and weight function *W* to assign weight to each edge. The ends of each contig are annotated by two tags, *B* and *E* where *B* stands for the beginning of the contig and *E* stands for the end of the contig. Using this concept of node, there are 4 types of edges in the graph, *BE* joining beginning of first contig to end of second, *EB* joining end of the first contig to beginning of second, *BB* joining beginning of the first contig to beginning of second contig and *EE* joining end of the first contig to end of second (Fig. 6 a). Using raw counts of Hi-C read pairs shared between ends of two contigs is not the correct way to score the edges because of length biases. Since two long contigs with a large genomic distance tend to share more read pairs compared to two short or one long and one short contig with much lesser genomic distance. To address this issue, we define an edge weight function in such a way that it reduces such length biases. We define a length cutoff *l* and consider the read pairs mapped in the region of length *l* at both ends (*B* and *E*) of contigs. Our edge weight normalization is based on how many times the restriction enzyme used in Hi-C protocol cuts the region of length *l* and divide the counts of read pairs by this number. Putting all this together, the edge weight function is expressed as:

$$W(E) = \frac{links(C1,C2)}{RE(C1) + RE(C2)} $$

where *C*1 and *C*2 are the contigs yielding the edge, *l* *i* *n* *k* *s*(*C*1,*C*2) is the number of Hi-C links present within the region of length *l* from the end of contigs and *R* *E*(*C*1) and *R* *E*(*C*2) is the number of sites cut by the restriction enzyme in the region *l* at the end of *C*1 and *C*2. This gives us the normalized edge weights which we use for scaffolding. Once we calculate the edge weights, we construct the graph *G* as follows. We first sort all the edges in decreasing order of their weights given by *W*. After this, we remove all the edges which have very low number of read pairs associated to them which mostly account for sequencing errors. Once edges are sorted and filtered, we construct *G* according to Algorithm 1. We greedily add edges to *G* only if both the nodes comprising an edge are not present in *G*. Finally, we add edges between *B* and *E* ends of same contigs to *G* which completes the graph construction. Figure 6 shows the intermediate steps involved in graph construction. In some cases, *G* can contain a cycle. Consider the following scenario. Suppose while constructing *G*, we add edges ‘ *X*:*B* - *Y*:*B*’ and ‘ *X*:*E* - *Y*:*E*’. After we scan through all the links, we add ‘ *X*:*B* - *X*:*E*’ and ‘ *Y*:*B* - *Y*:*E*’ edges to *G*. This creates a cycle of length 4 in *G*. However, this kind of cycle can easily be removed by removing the lower cost edge among *BB* and *EE* edges. Once we remove cycles from *G*, we get the final scaffold graph which we use for further analysis.

### Scaffold construction

Before explaining the scaffold construction algorithm, we prove following lemmas to understand the properties of *G*.

#### **Lemma 1**

*G* has no nodes with degree greater than *2*.

#### *Proof*

While constructing *G*, we add edges at most twice for each node. First when we have no edge associated to that node and second when we add an edge between *B* and *E* ends of the contig associated with that node. If some node has degree greater than 2, it would mean that we added an edge to that node apart from the cases described previously, which is a contradiction. □

#### **Lemma 2**

Each connected component of *G* has exactly two nodes of degree *1*.

#### *Proof*

We know from the construction of *G* that *G* has no cycles. We can prove this for some connected component *C* of *G* and the argument can be applied to all connected components. Since *G* has no cycles, *C* will have at least one node with degree 1. In the first case, suppose *C* has exactly one node of degree 1. This implies that we have at least two edges originating from all other nodes in *C*. It would mean that there exists at least one node in *C* with a degree at least 3. This is a contradiction because of Lemma 1. In the second case, suppose *C* has more than two nodes of degree 1. It would mean that there exists at least one node in *C* with degree 3. This is again a contradiction due to Lemma 1. □

Knowing these properties about *G* we construct scaffolds as described in Algorithm 2. First, a threshold *N*_*th*_ is decided for a scaffold to qualify as a seed scaffold. If a scaffold has a number of contigs greater than *N*_*th*_, it is marked as seed scaffold. For each connected component of *G*, we first find out two nodes *u* and *v* with degree 1. We will always find such nodes due to Lemma 2. After this, the path connecting *u* and *v* is found in the connected component. In the context of *G*, we define a path as an alternating sequence of nodes and edges. The edges can be either between the ends of same contigs (contig edge) or the ends of different contigs (Hi-C implied edge). Since all the nodes in the connected component have degree either 1 or 2, there will always be just 1 path connecting *u* and *v*. It can also be observed from Lemma 1 and 2 that, all other nodes in the connected component lie on the path connecting *u* and *v*. If this path has the number of contigs greater than *N*_*th*_, we mark this path as a seed scaffold, otherwise, it is marked as a small scaffold.

Even after edge weight normalization, there can still exist some length biases, omitting some of the small contigs from the seed scaffolds. To account for this, we develop a method to insert the contigs in small scaffolds into seed scaffolds. First, for each contig in small scaffolds, exactly one seed scaffold is assigned to it based on the total edge weight of the edges in the original scaffold graph connecting this contig to all the contigs in seed scaffold. After this, each contig is tested for insertion into its corresponding seed scaffolds in both the orientations at all possible position. It is inserted at the position where it maximizes the total weight of the scaffold. Once all the contigs are inserted into seed scaffolds, it leaves us with the final scaffolds. The algorithm is sketched in Algorithm 2.



## Additional file

Additional file 1 Supplementary Figures. Figures for dot plots for each chromosomes of NA12878 for LACHESIS and SALSA scaffolds, dot plots for scaffolds of goat optical map scaffolds and coverage plot for misassembled contig. (DOCX 664 kb)

## Abbreviations

BWA: Burrows wheeler aligner
RH map: Radiation Hybrid map
SALSA: Simple assembly scaffolder
SMRT: Single molecule real time
WDAG: Weighted directed acyclic graph
