# Supplementary material for: Scaffolding of long read assemblies using long range contact information
Source: BMC Genomics. 2017 Jul 12;18:527. doi: 10.1186/s12864-017-3879-z (PMC5508778; doi:10.1186/s12864-017-3879-z)
Supplement: Additional file 1 — Supplementary Figures. Figures for dot plots for each chromosomes of NA12878 for LACHESIS and SALSA scaffolds, dot plots for scaffolds of goat optical map scaffolds and coverage plot for misassembled contig. (DOCX 664 kb) [file 12864_2017_3879_MOESM1_ESM.docx]

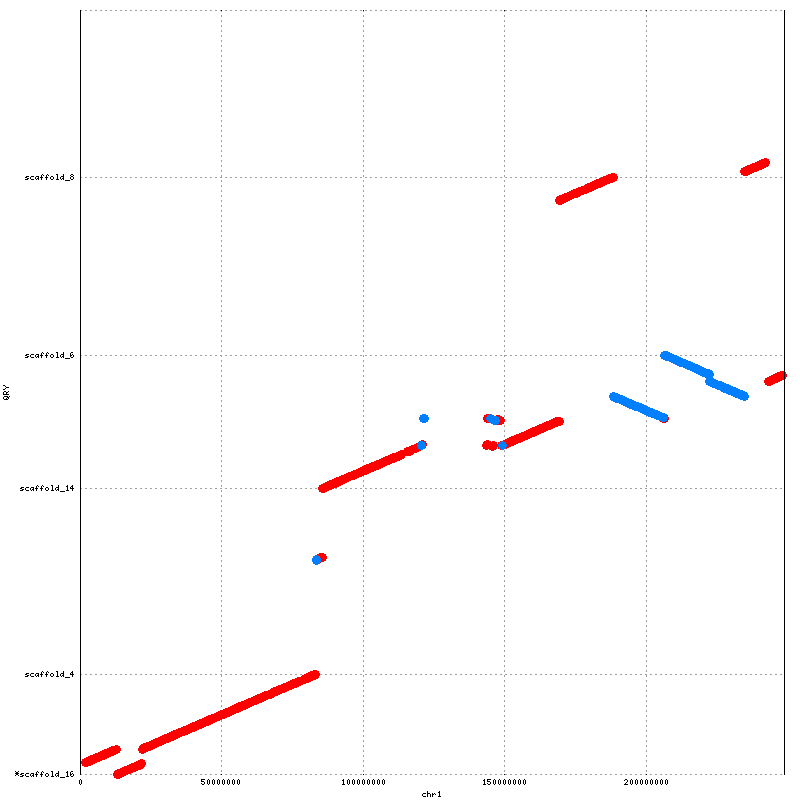

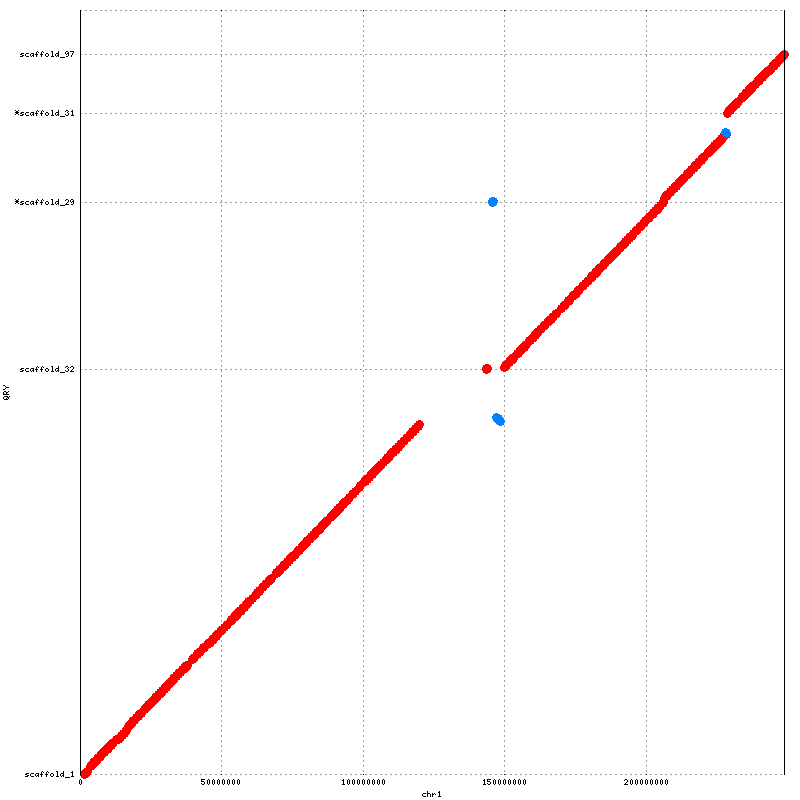
[\\\\\\\\\\\||\E](file:///\\\\\\\\\\\||\E)


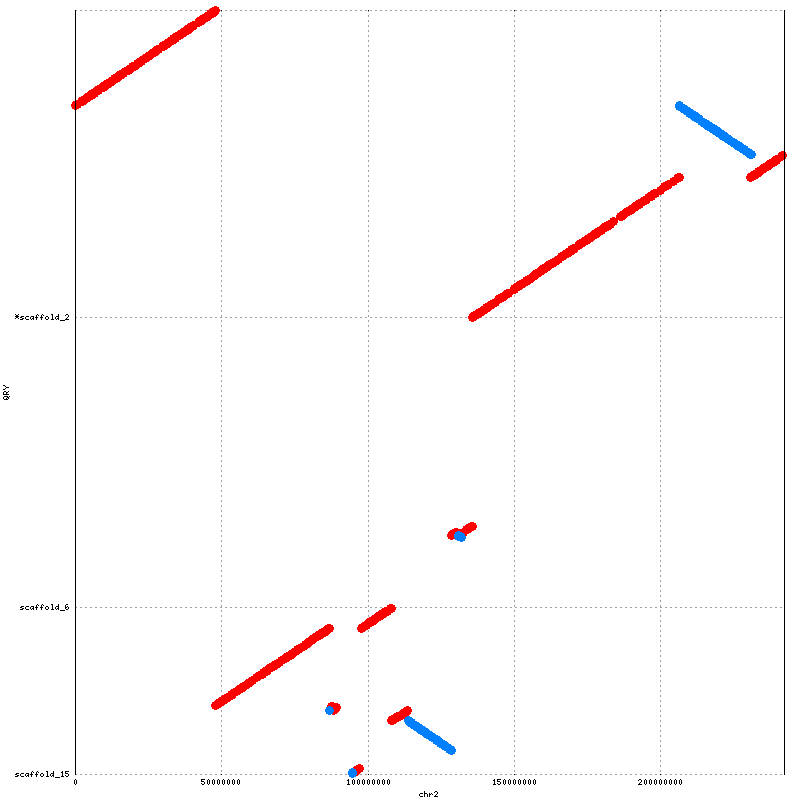

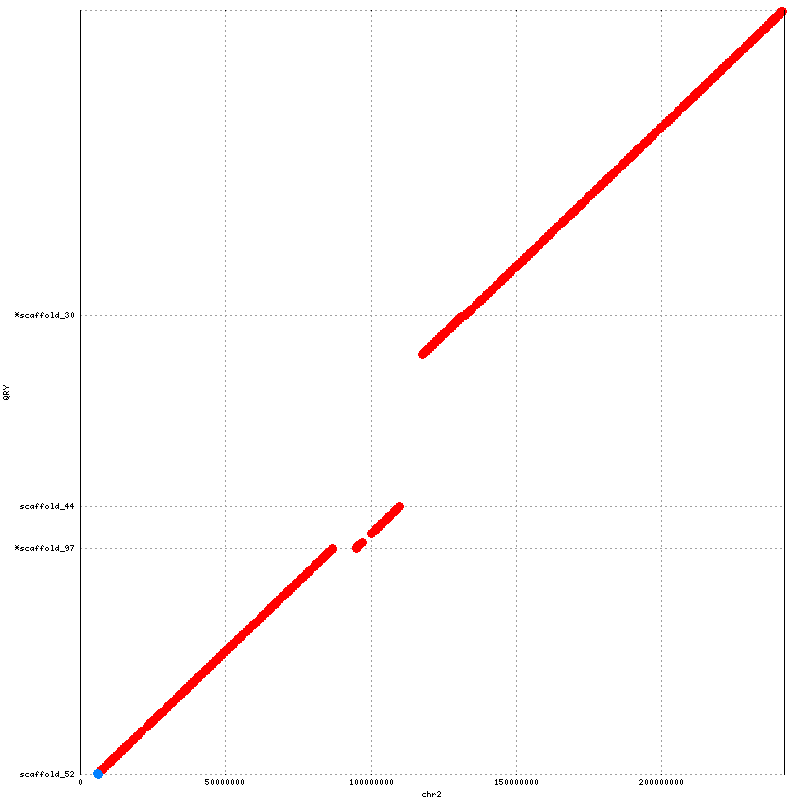


Supplementary Figure S1: Orientation and ordering results for SALSA (on the left) with Lachesis (on the right) for NA12878. These plots are in detailed view for each chromosome in Figure 3

**Chr2**

**Chr2**

**Chr1**

**Chr1**


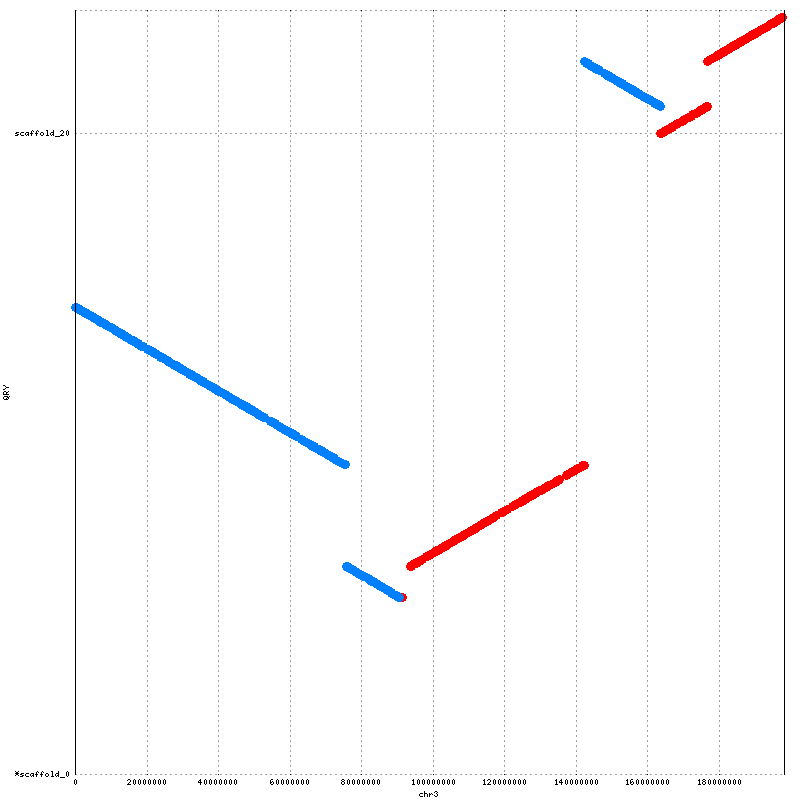

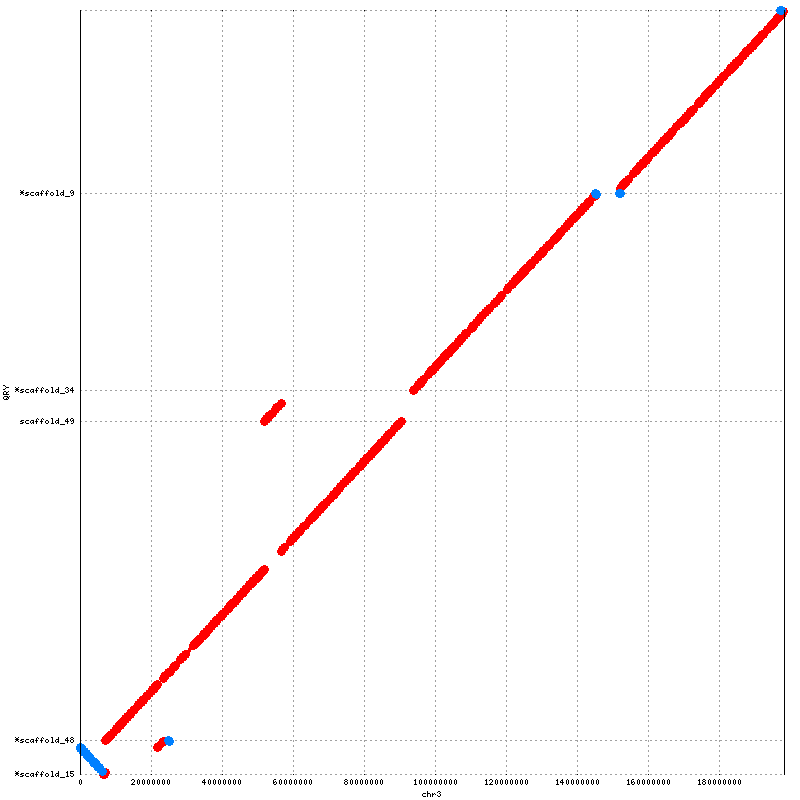


**Chr3**

**Chr3**


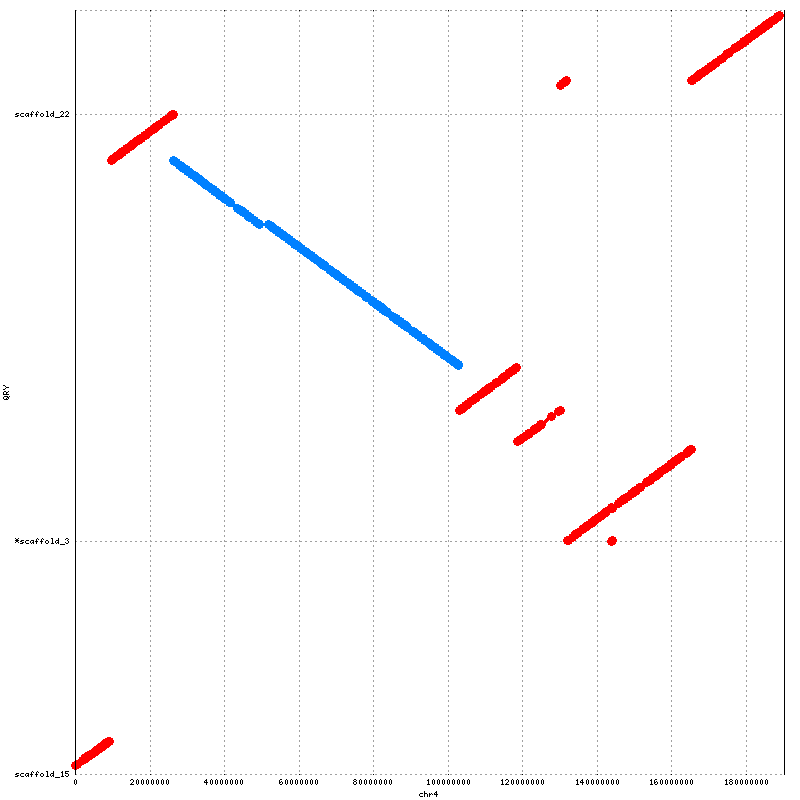

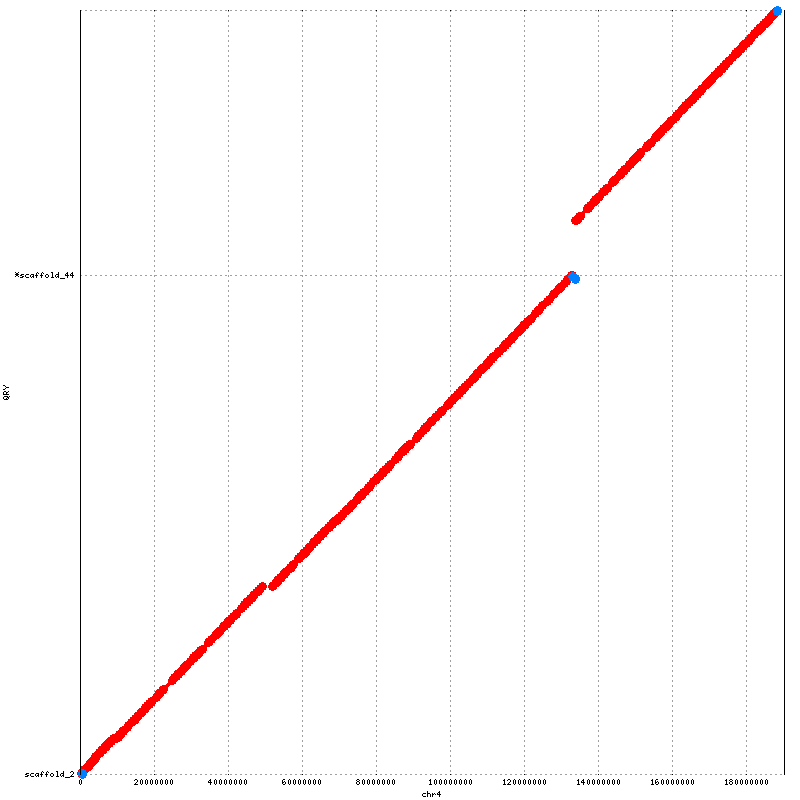


**Chr4**

**Chr4**

Supplementary Figure S1: Orientation and ordering results for SALSA (on the left) with Lachesis (on the right) for NA12878. These plots are in detailed view for each chromosome in Figure 3


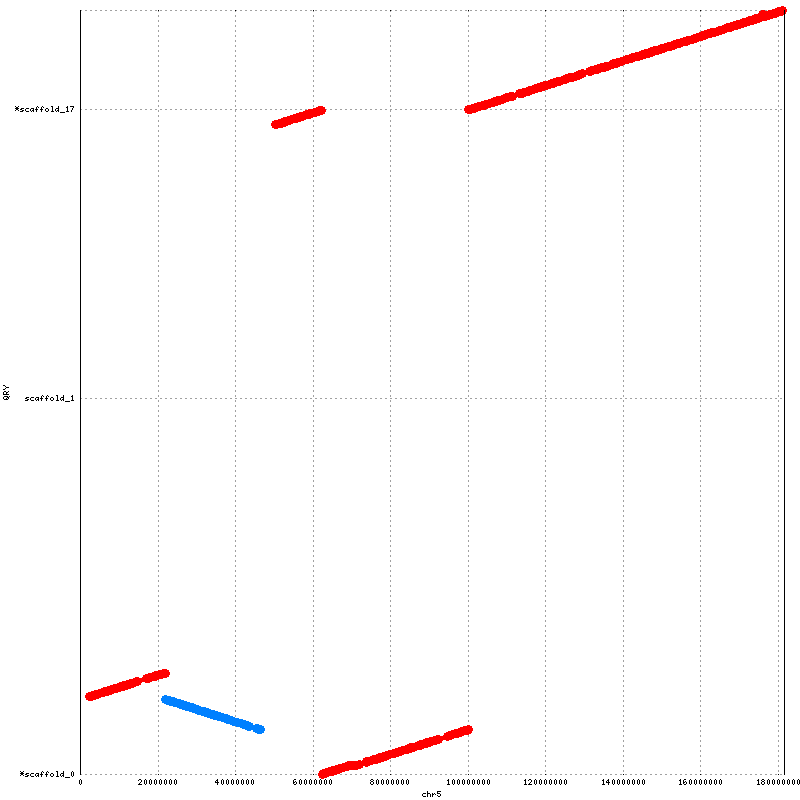

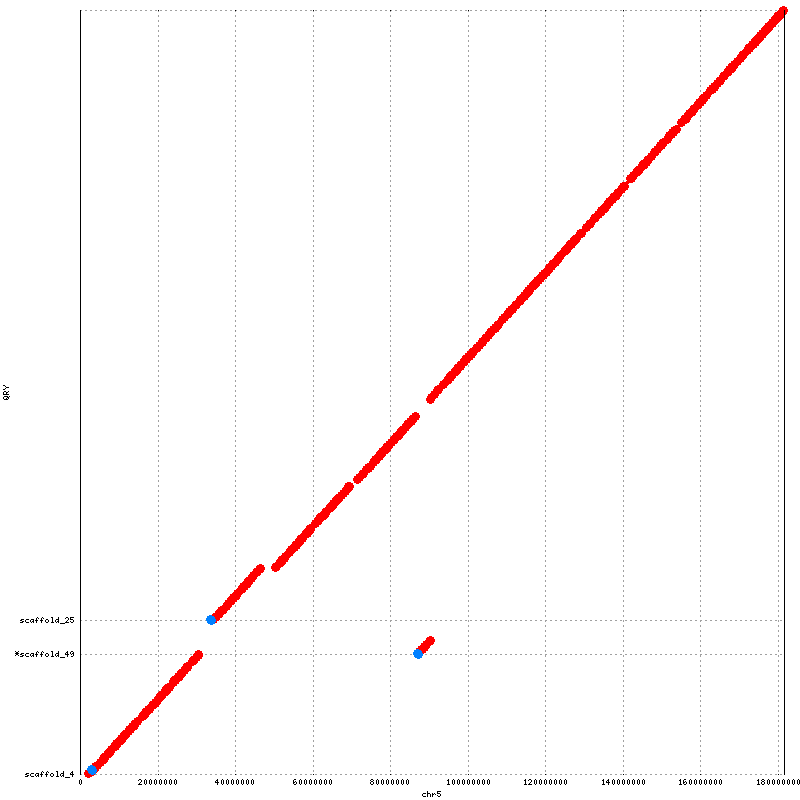


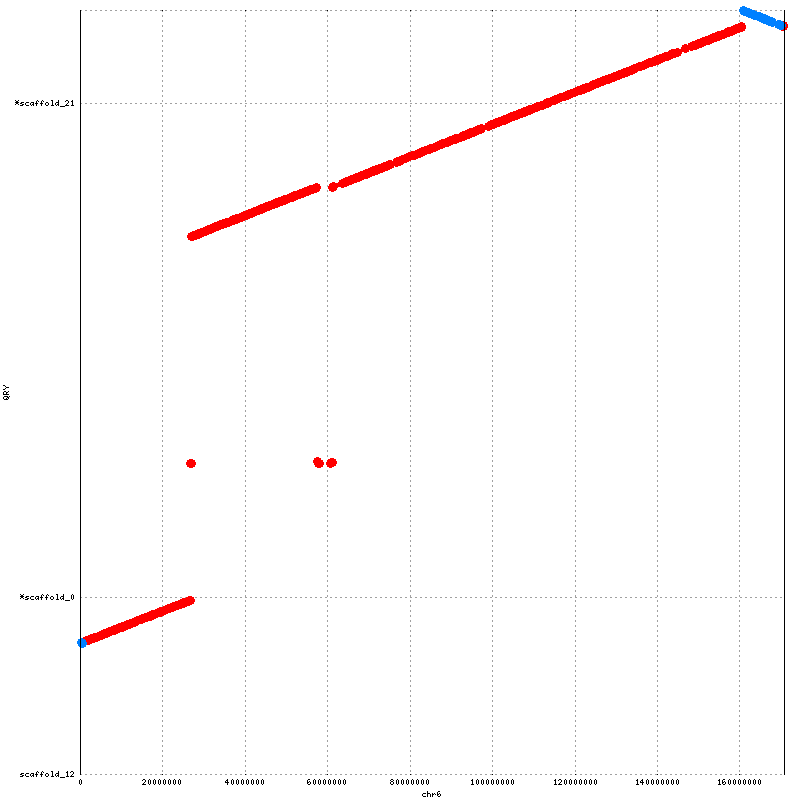

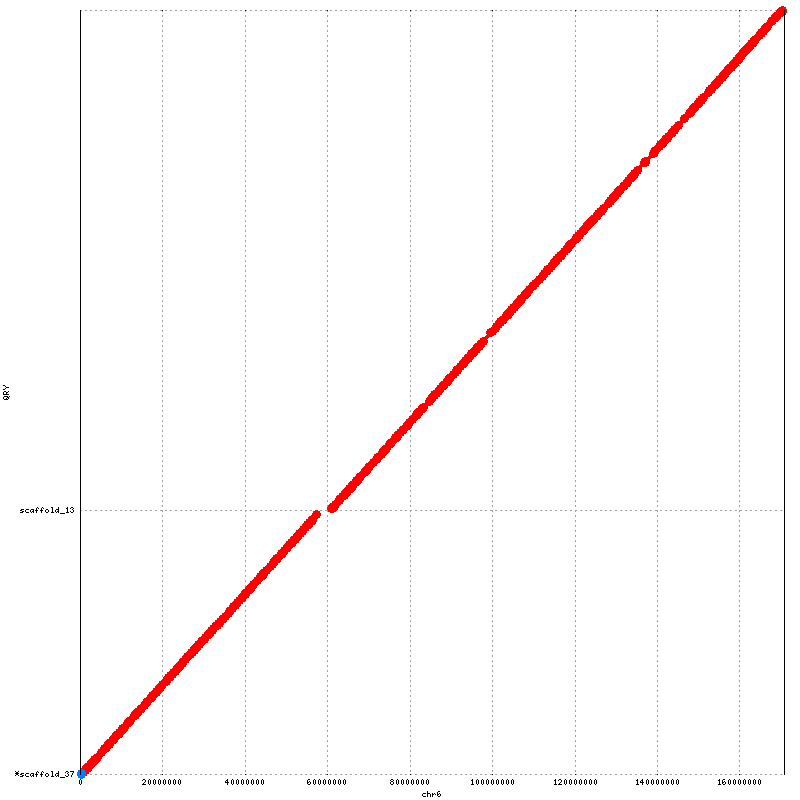


Supplementary Figure S1: Orientation and ordering results for SALSA (on the left) with Lachesis (on the right) for NA12878. These plots are in detailed view for each chromosome in Figure 3

**Chr6**

**Chr6**

**Chr5**

**Chr5**


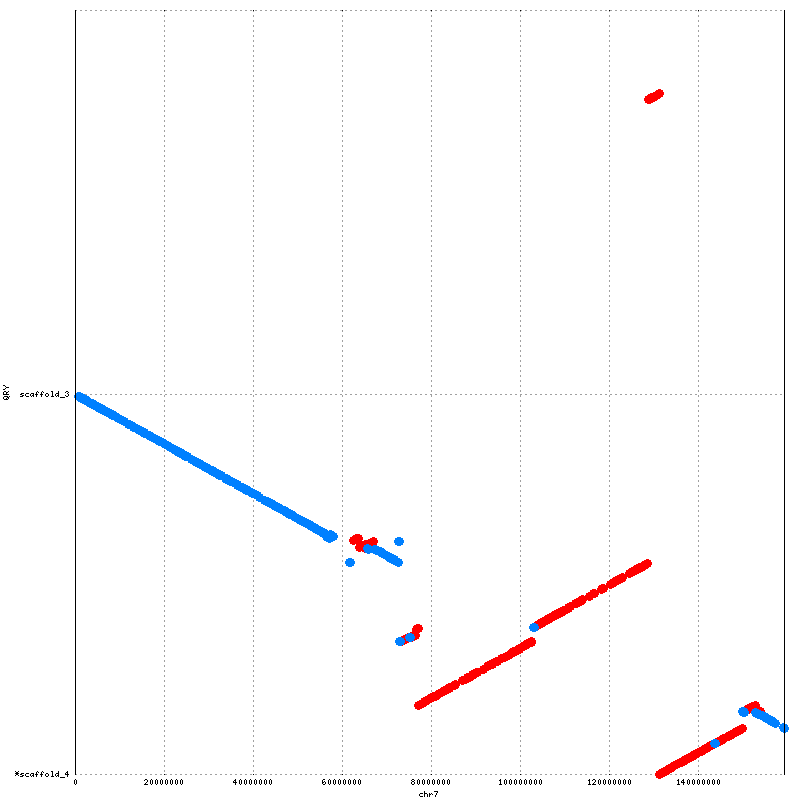

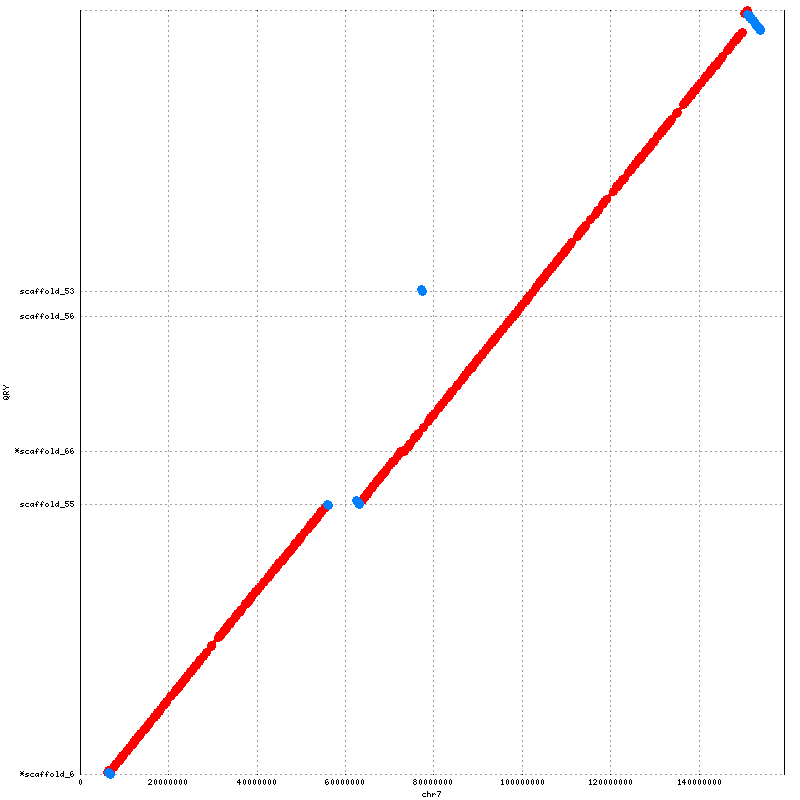


**Chr7**

**Chr7**


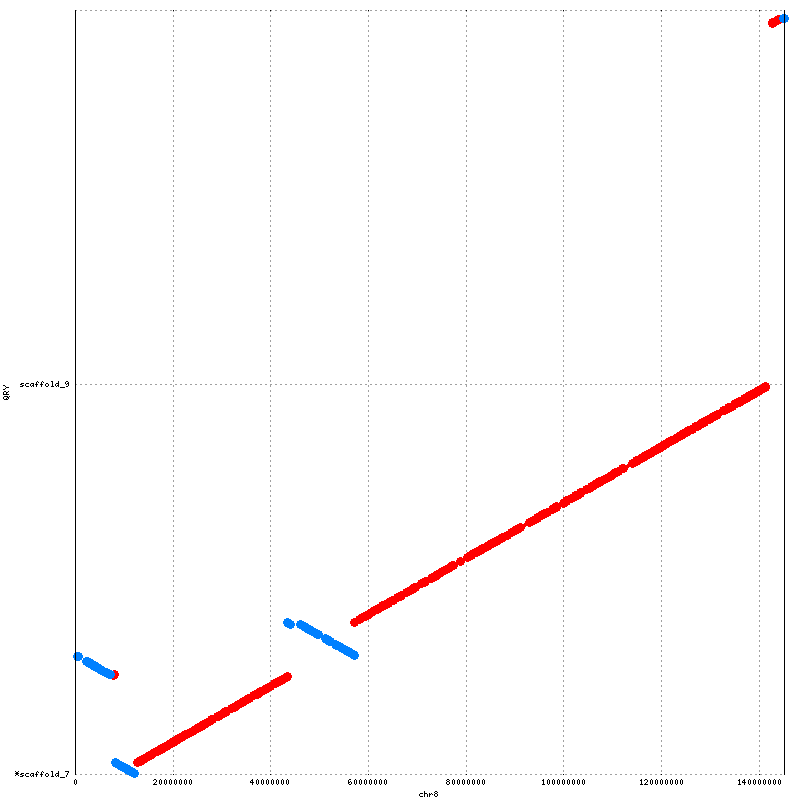

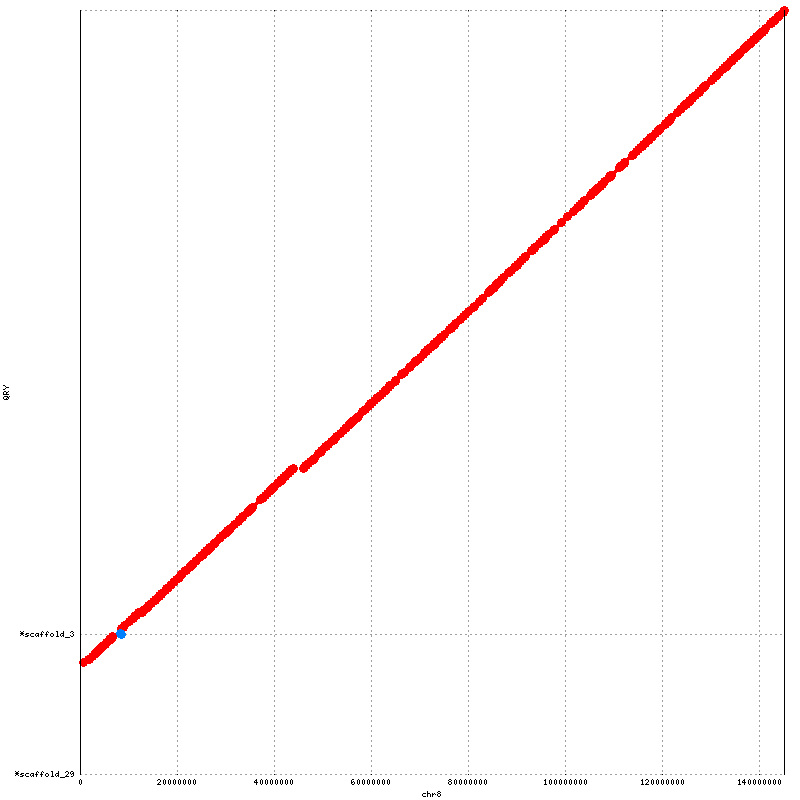


**Chr8**

**Chr8**

Supplementary Figure S1: Orientation and ordering results for SALSA (on the left) with Lachesis (on the right) for NA12878. These plots are in detailed view for each chromosome in Figure 3


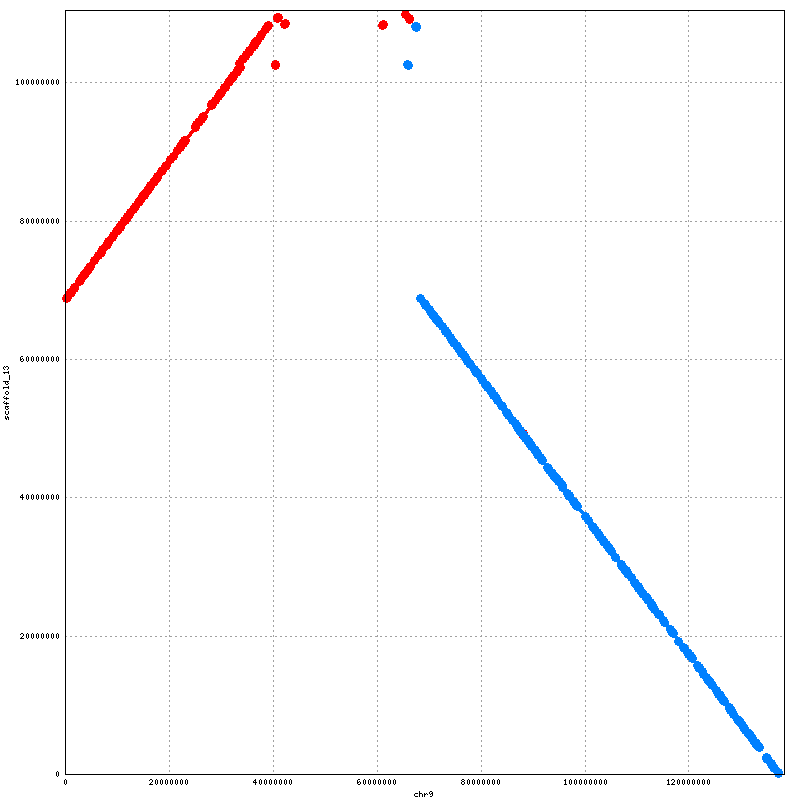

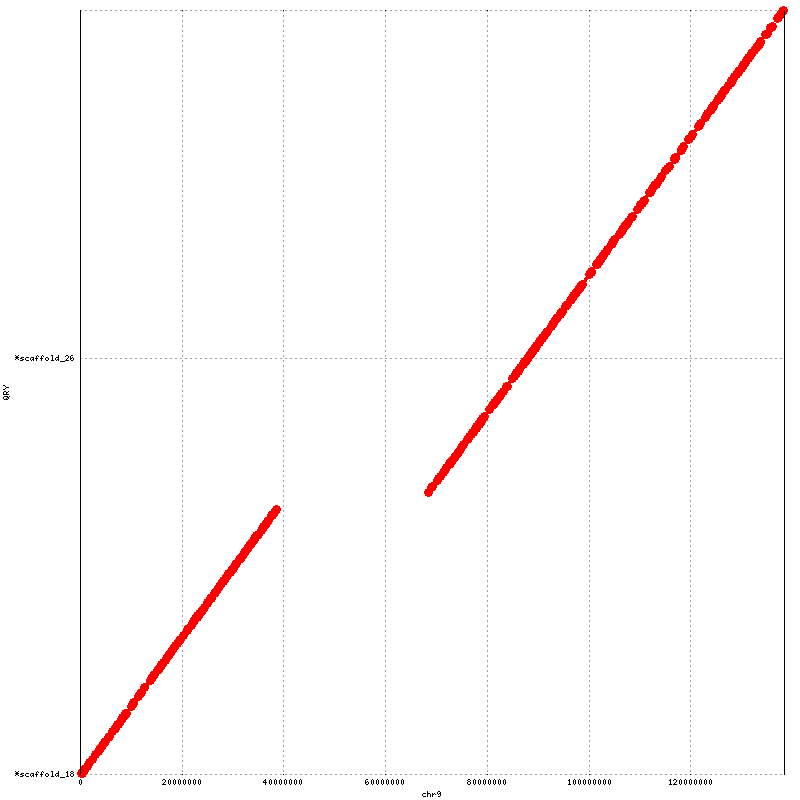


**Chr9**

**Chr9**


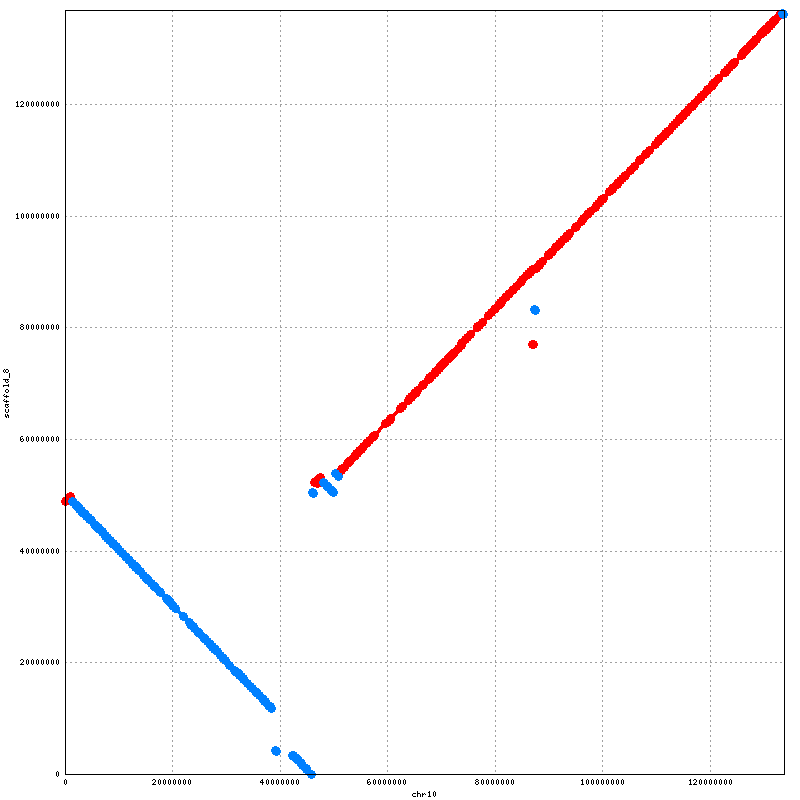

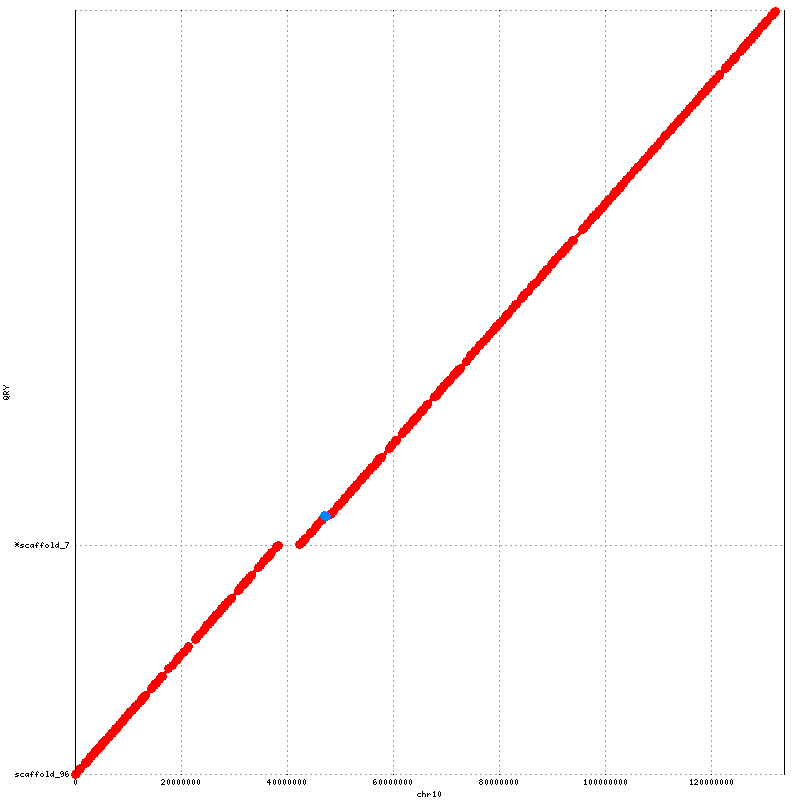


Supplementary Figure S1: Orientation and ordering results for SALSA (on the left) with Lachesis (on the right) for NA12878. These plots are in detailed view for each chromosome in Figure 3

**Chr10**

**Chr10**


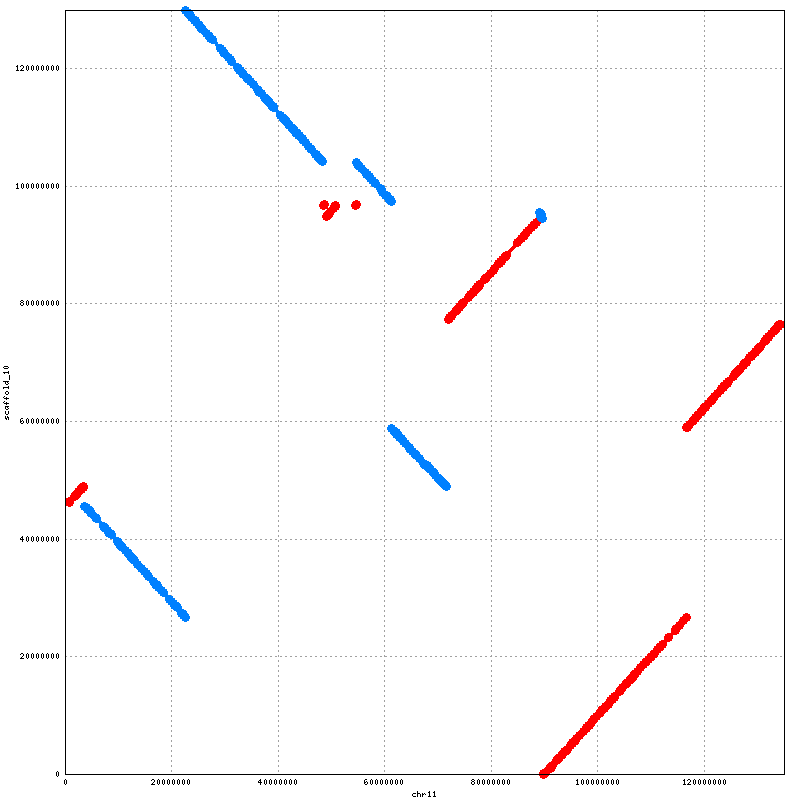

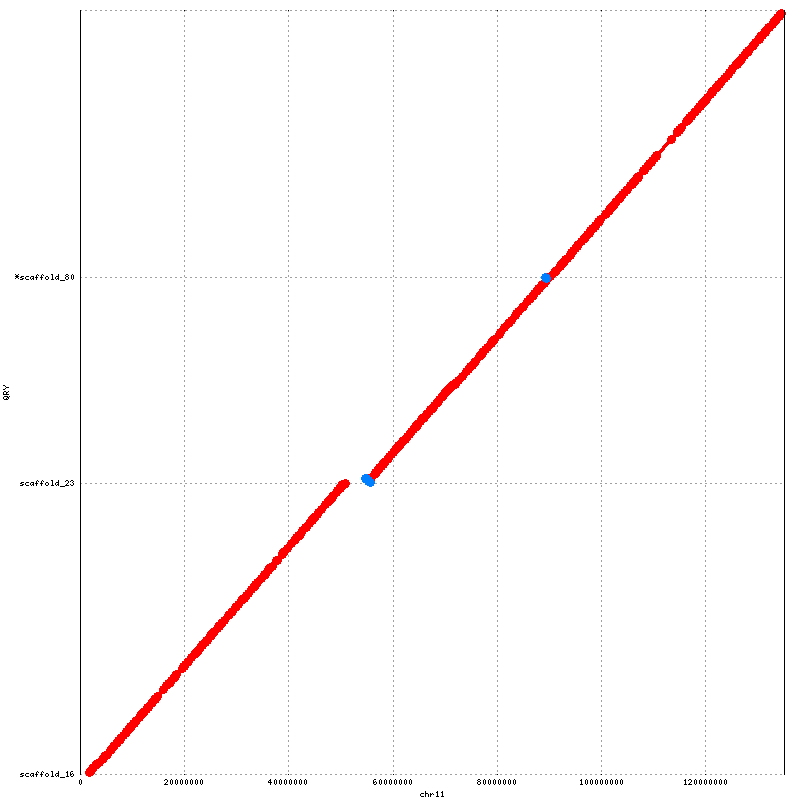


**Chr11**

**Chr11**


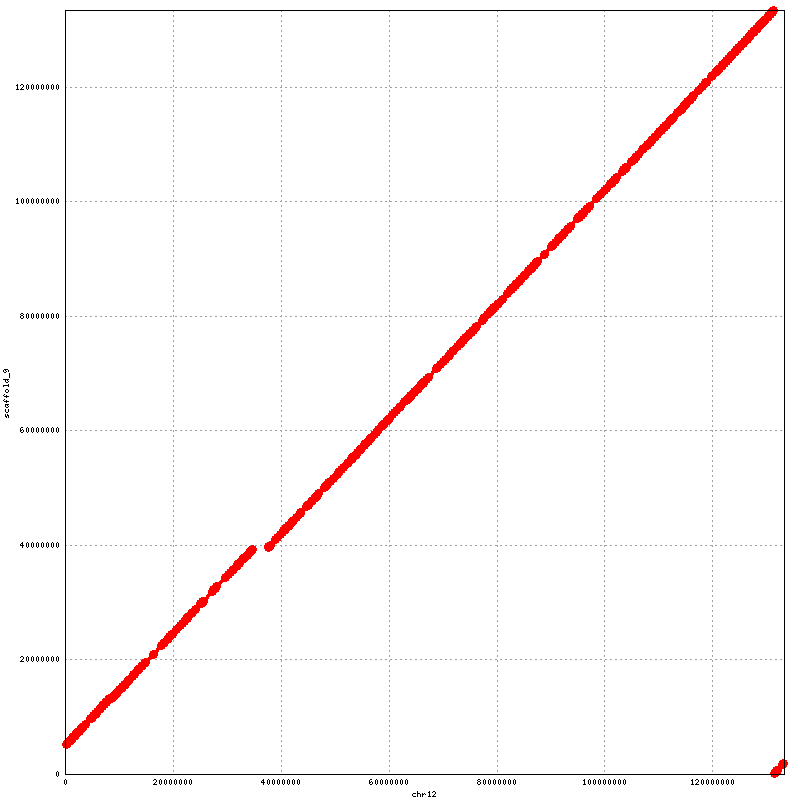

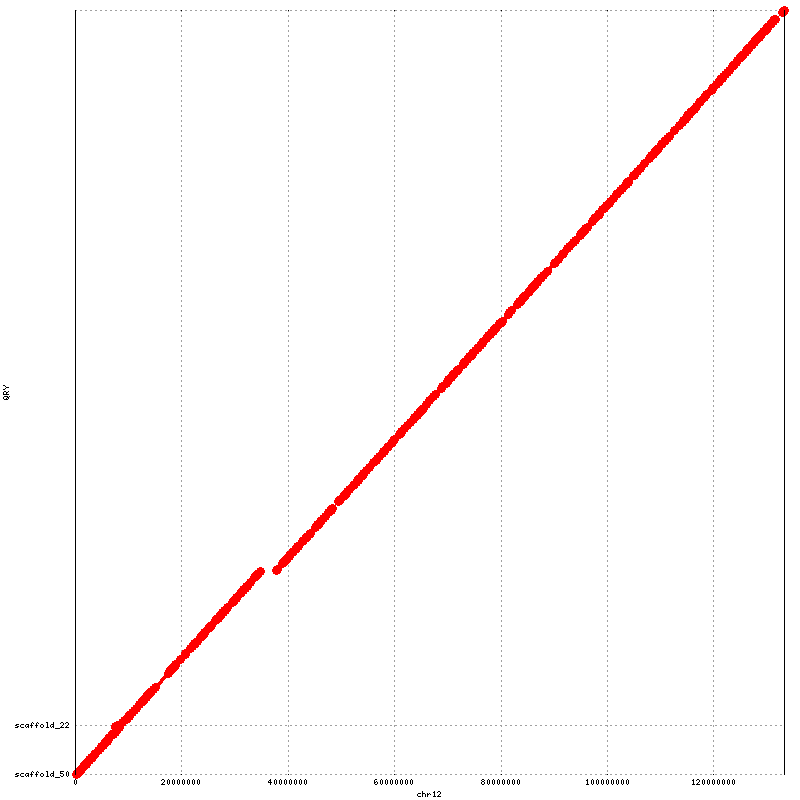


**Chr12**

**Chr12**

Supplementary Figure S1: Orientation and ordering results for SALSA (on the left) with Lachesis (on the right) for NA12878 . These plots are in detailed view for each chromosome in Figure 3


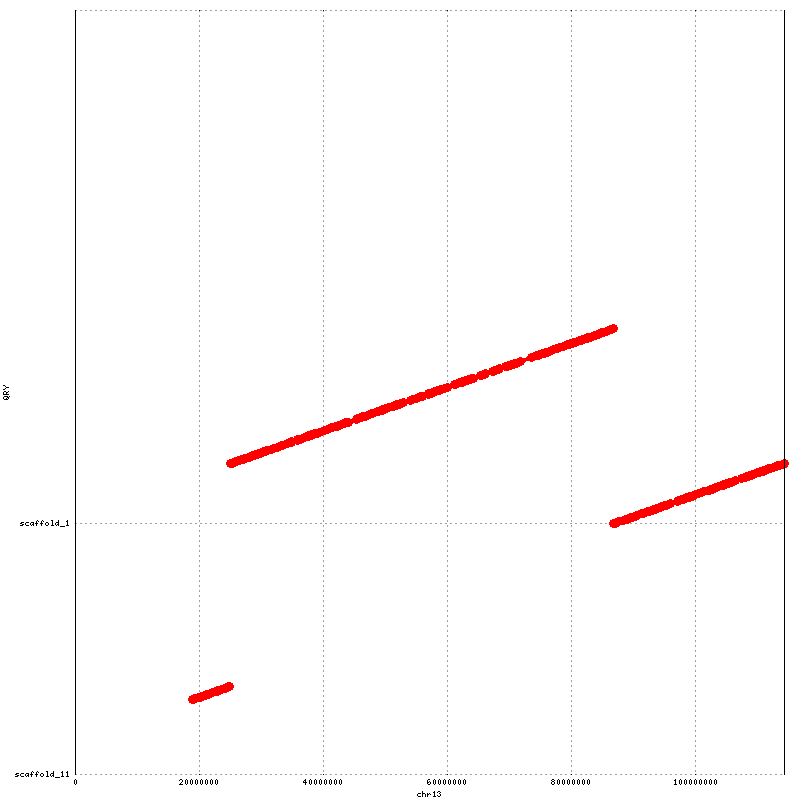

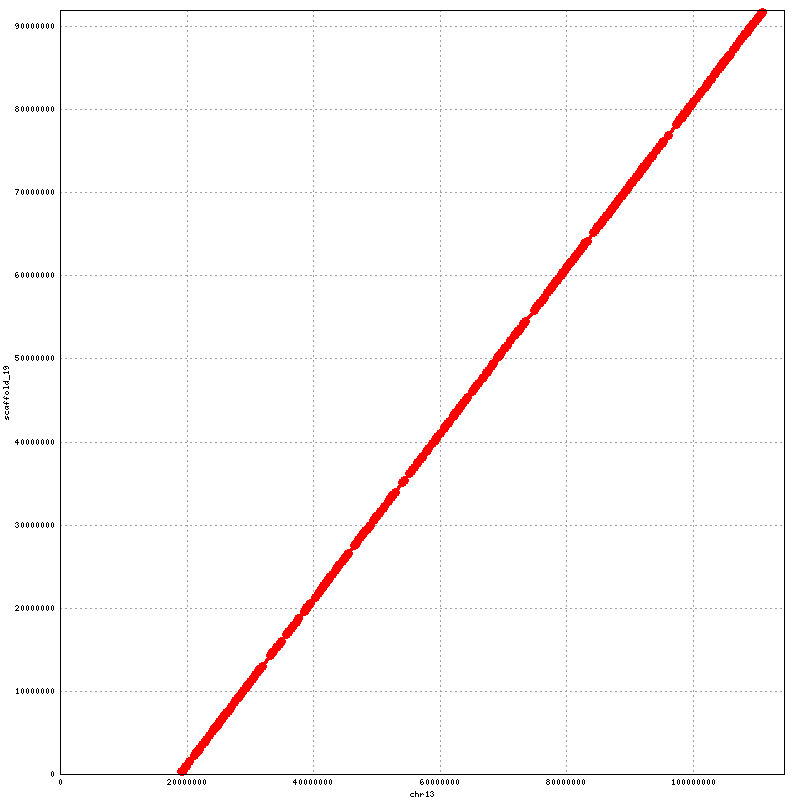


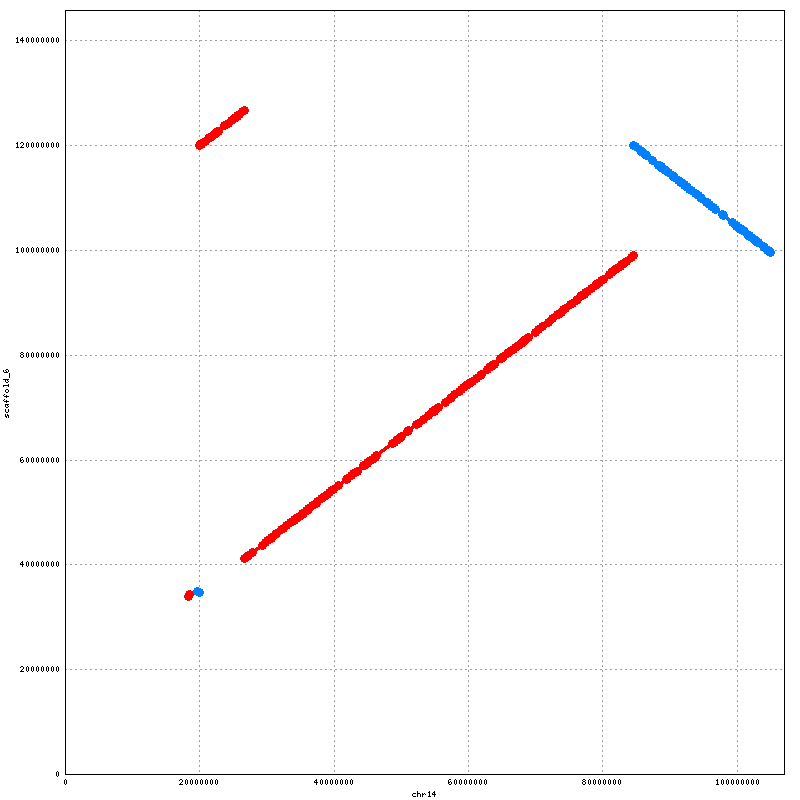

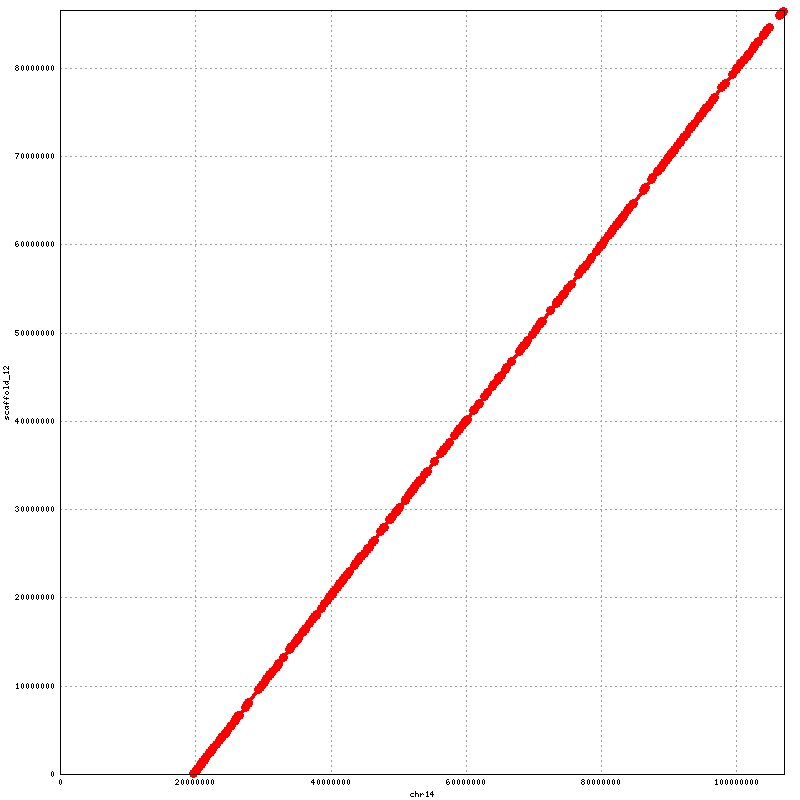


Supplementary Figure S1: Orientation and ordering results for SALSA (on the left) with Lachesis (on the right) for NA12878. These plots are in detailed view for each chromosome in Figure 3

**Chr14**

**Chr14**

**Chr13**

**Chr13**


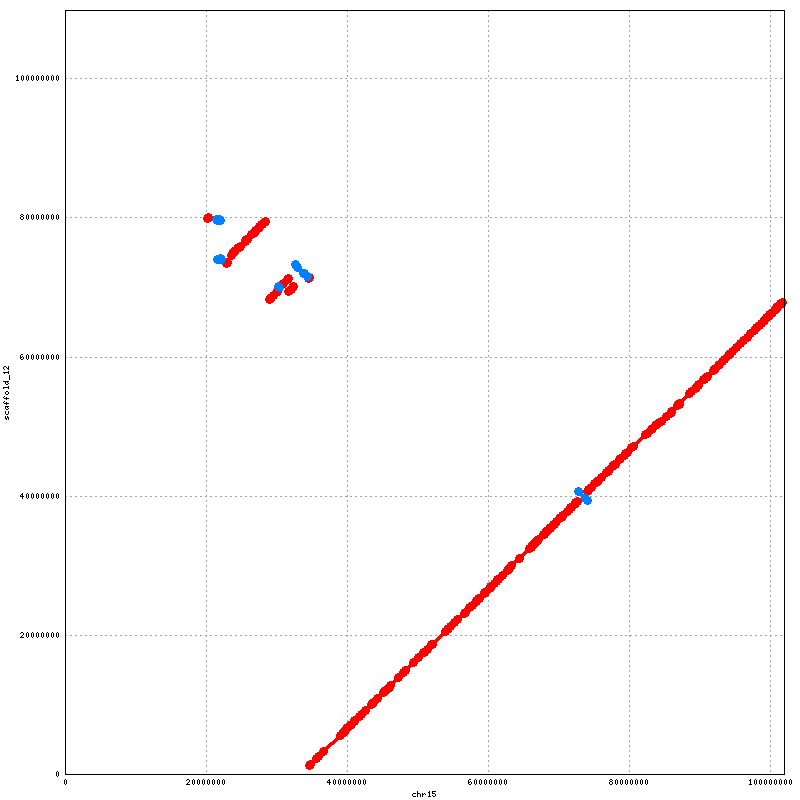

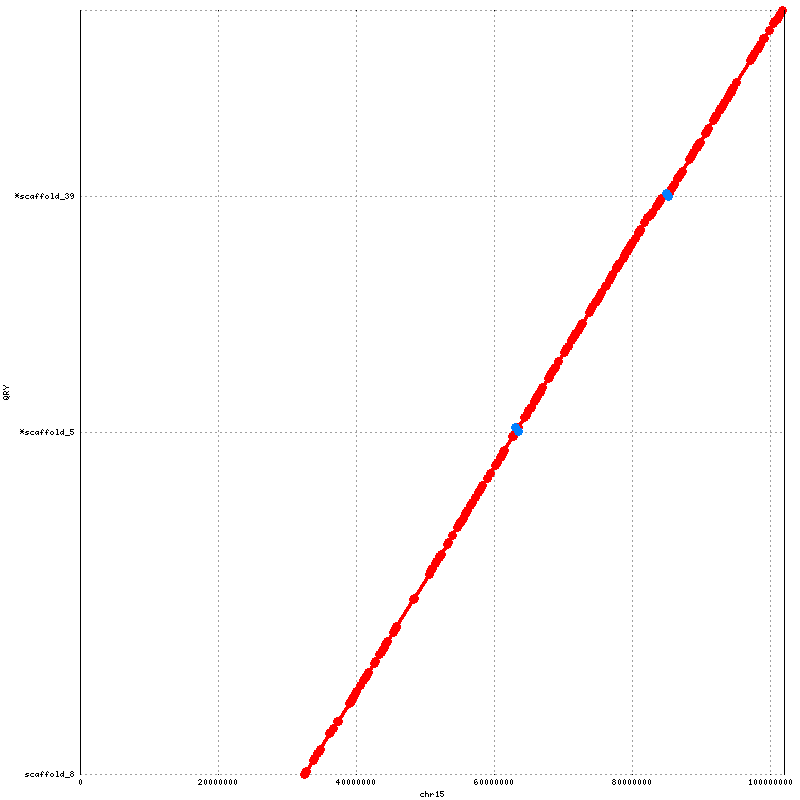
ß


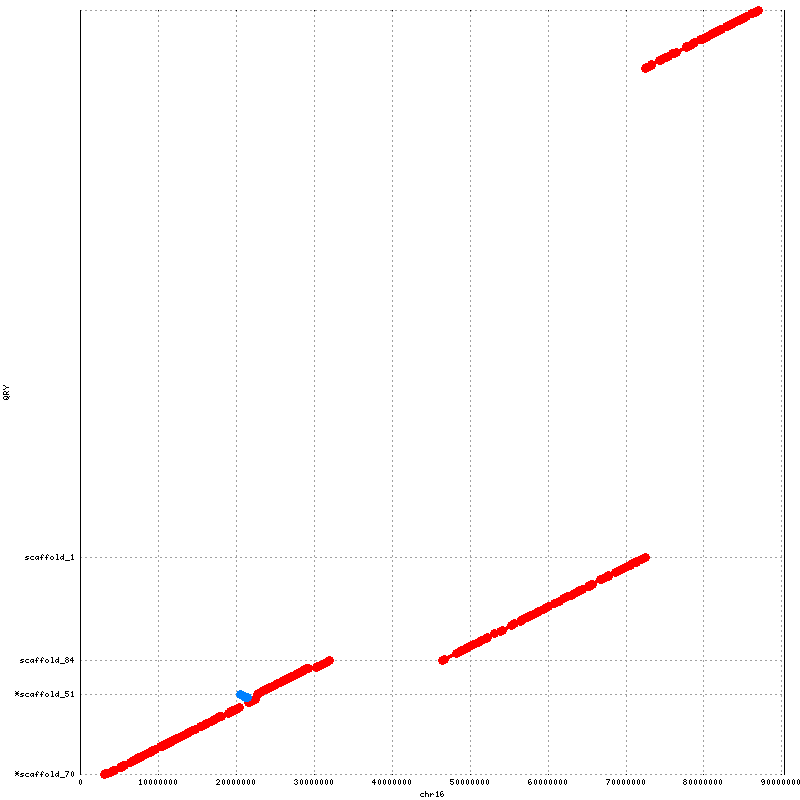

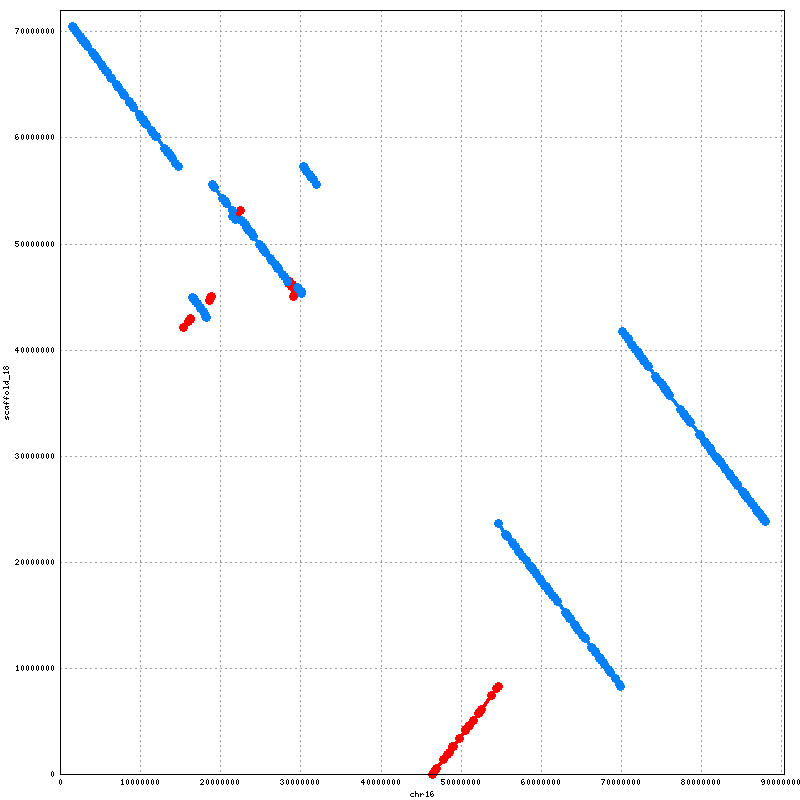


Supplementary Figure 1: Orientation and ordering results for SALSA (on the left) with Lachesis (on the right) for NA12878. These plots are in detailed view for each chromosome in Figure 3

**Chr16**

**Chr16**

**Chr15**

**Chr15**


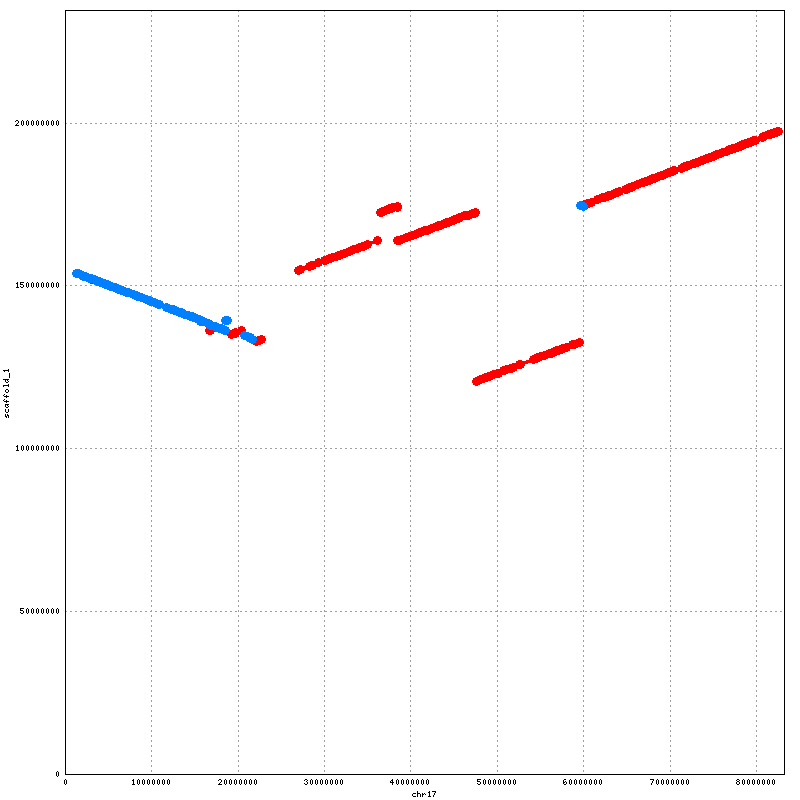

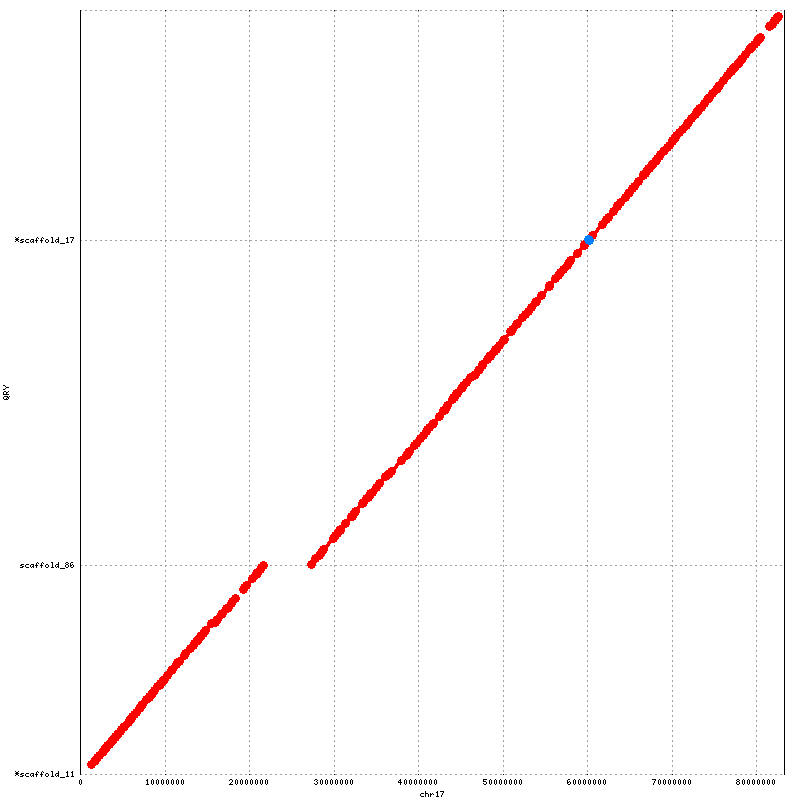


**Chr17**

**Chr17**


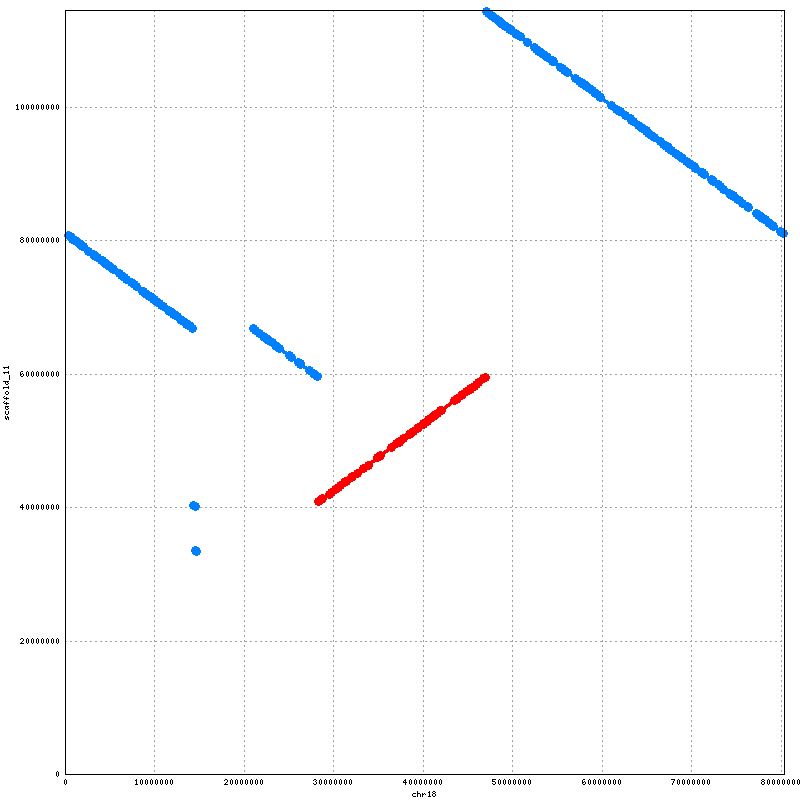

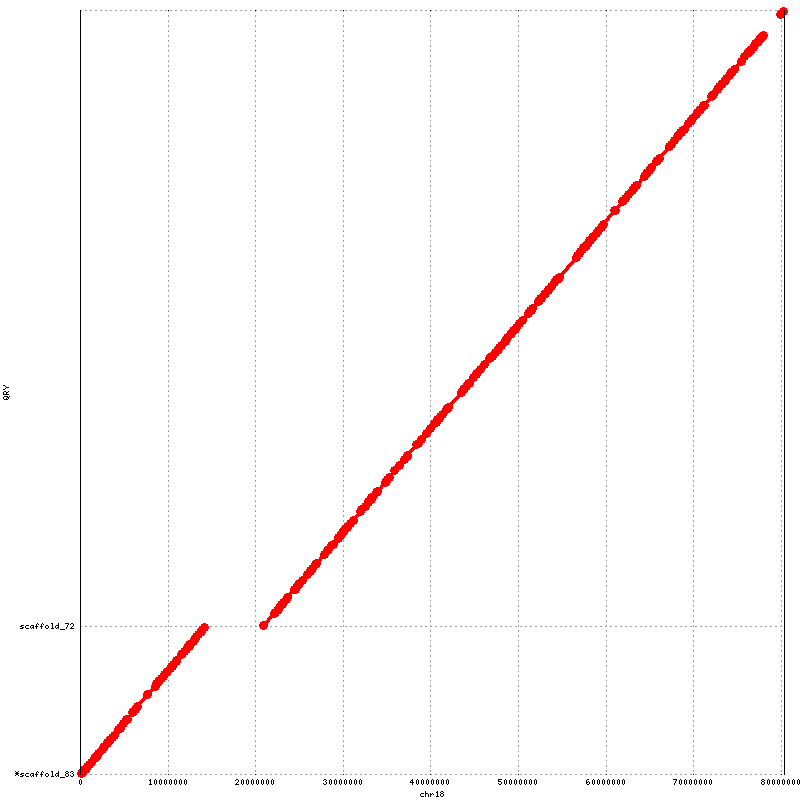


**Chr18**

**Chr18**

Supplementary Figure S1: Orientation and ordering results for SALSA (on the left) with Lachesis (on the right) for NA12878. These plots are in detailed view for each chromosome in Figure 3


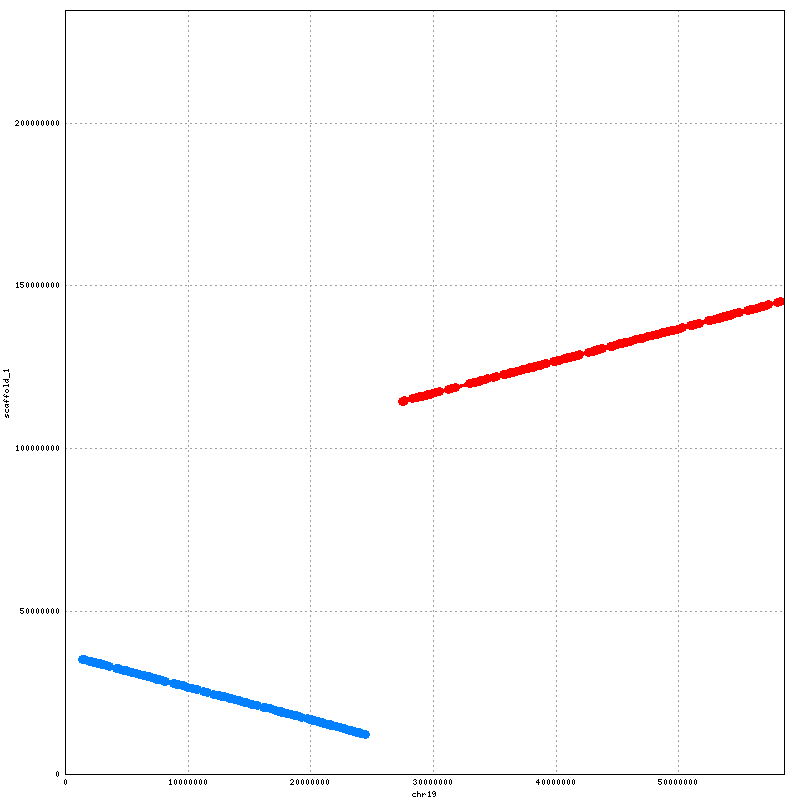

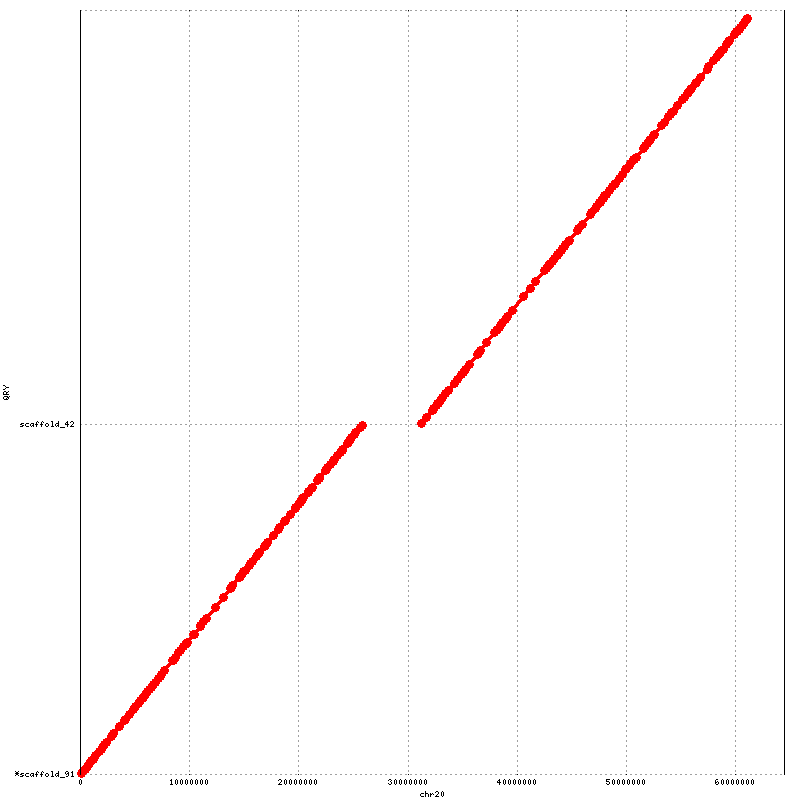

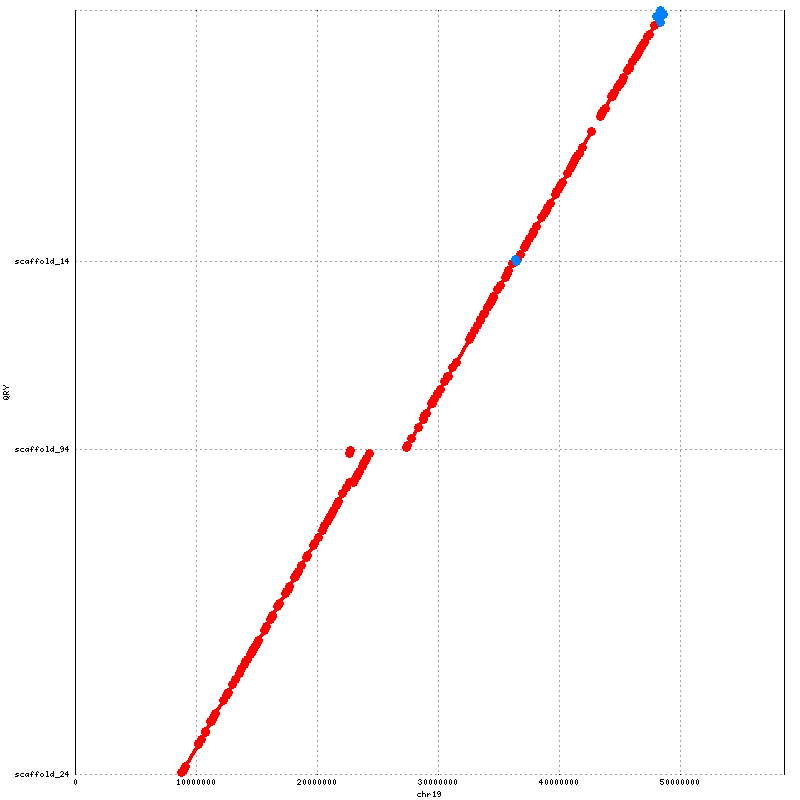


**Chr19**

**Chr19**


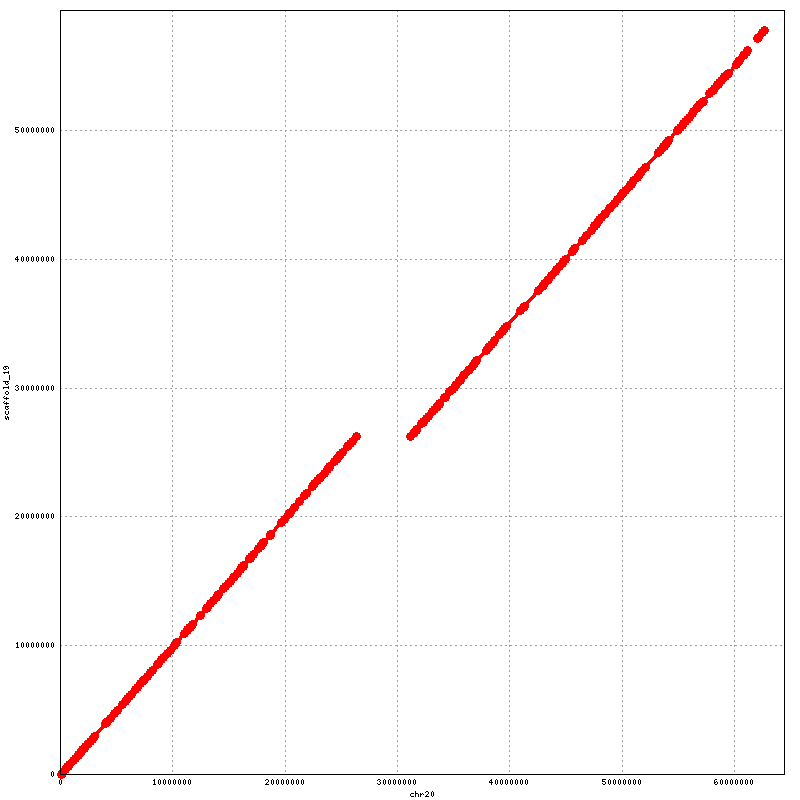


**Chr20**

**Chr20**

Supplementary Figure S1: Orientation and ordering results for SALSA (on the left) with Lachesis (on the right) for NA12878. These plots are in detailed view for each chromosome in Figure 3


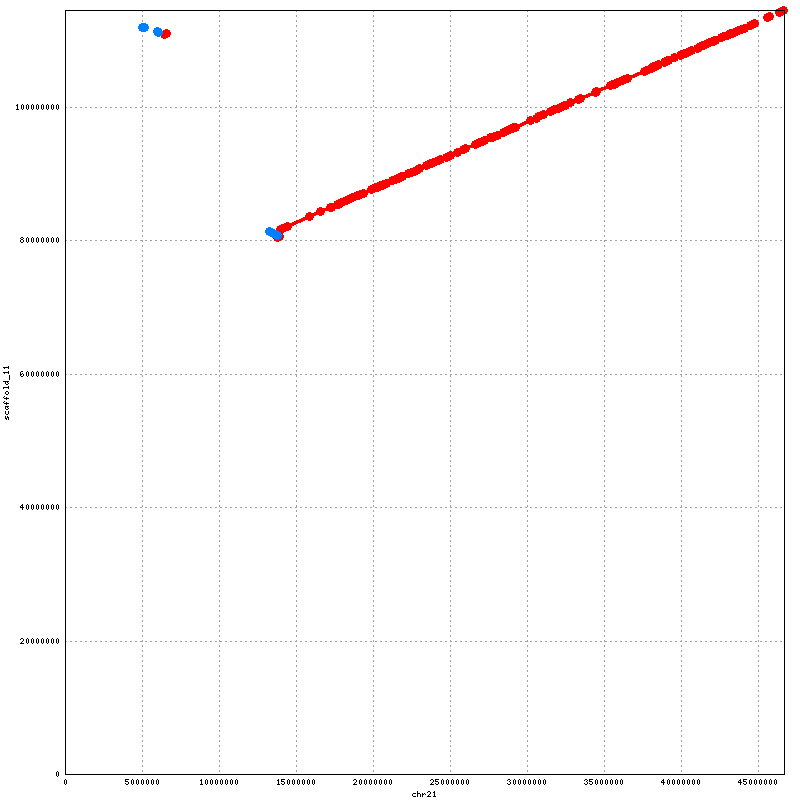

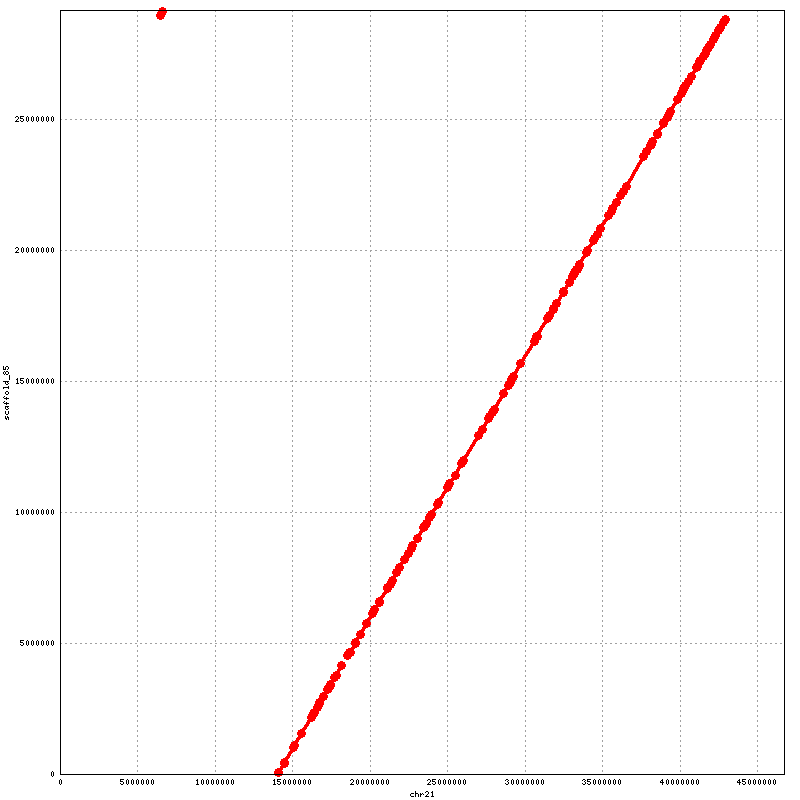


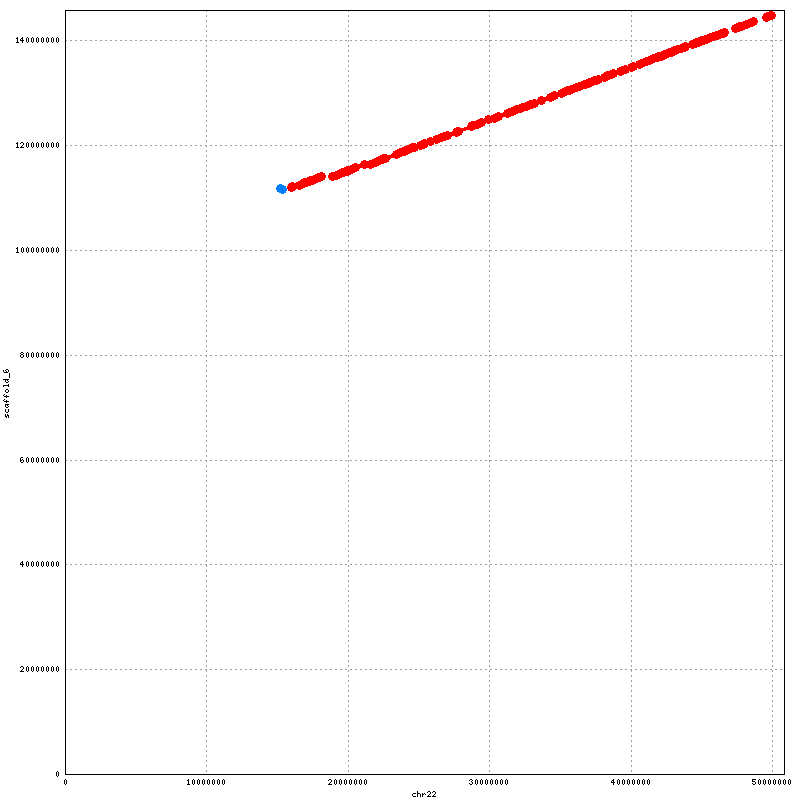

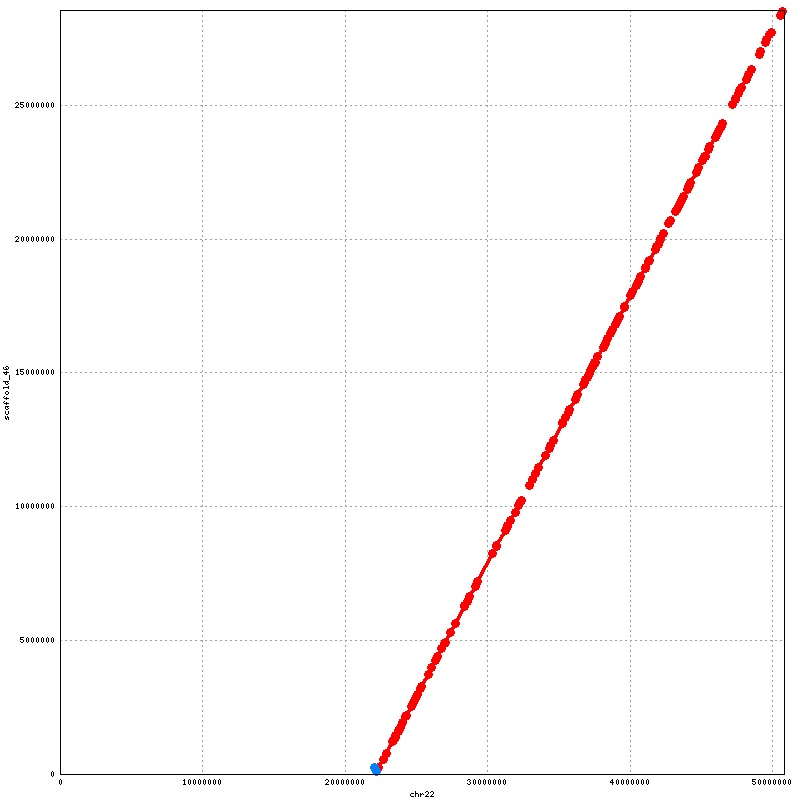


Supplementary Figure S1: Orientation and ordering results for SALSA (on the left) with Lachesis (on the right) for NA12878. These plots are in detailed view for each chromosome in Figure 3

**Chr22**

**Chr22**

**Chr21**

**Chr21**


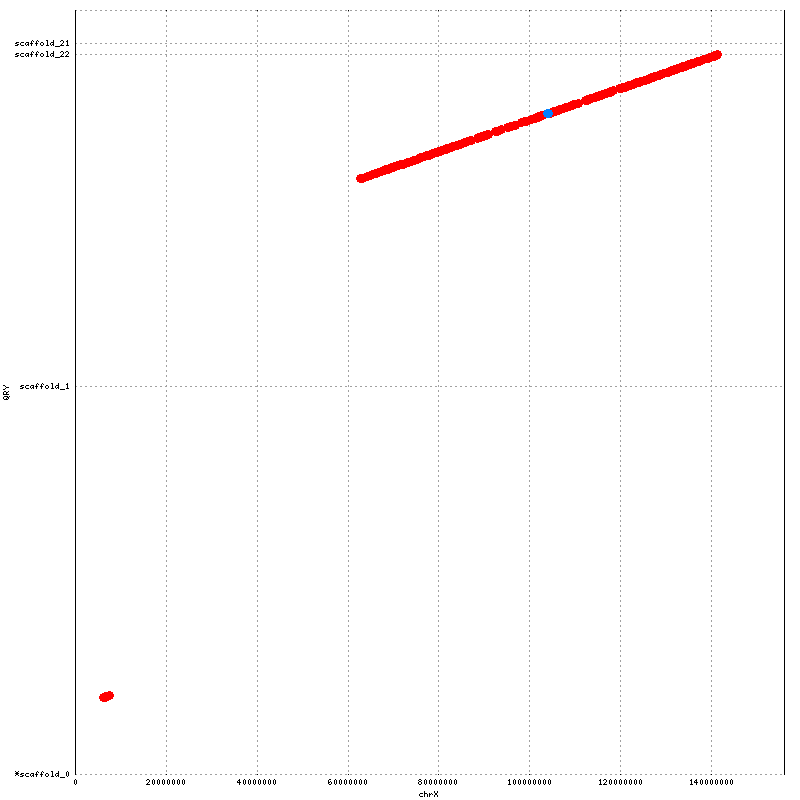

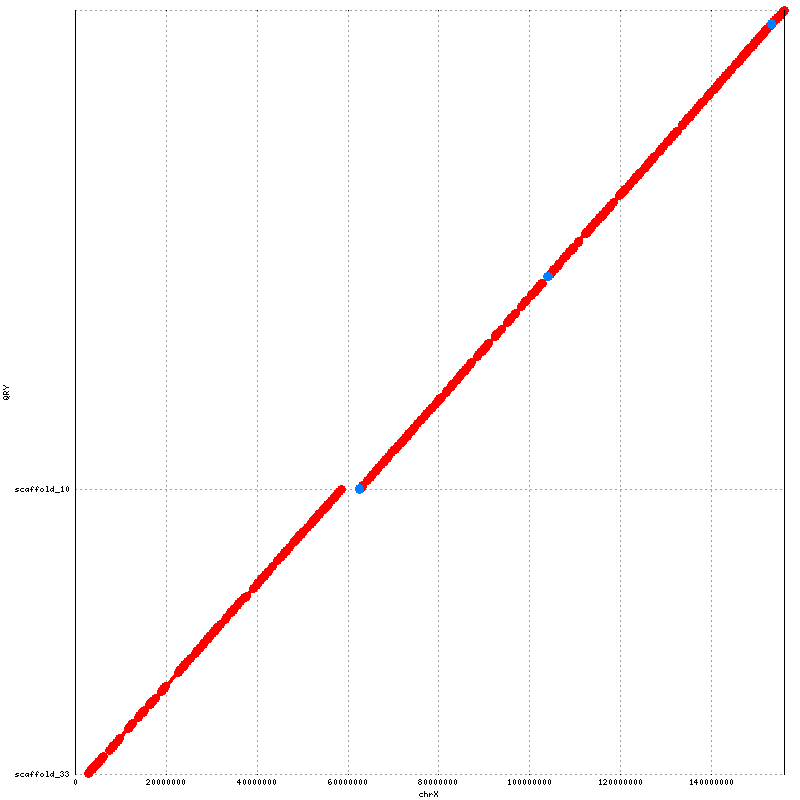


**ChrX**

**ChrX**

Supplementary Figure S1: Orientation and ordering results for SALSA (on the left) with Lachesis (on the right) for NA12878. These plots are in detailed view for each chromosome in Figure 3

Supplementary Figure S2: Alignment dot plots for scaffolds generated using bionano scaffolds by SALSA (on the left) and LACHESIS (on the right)


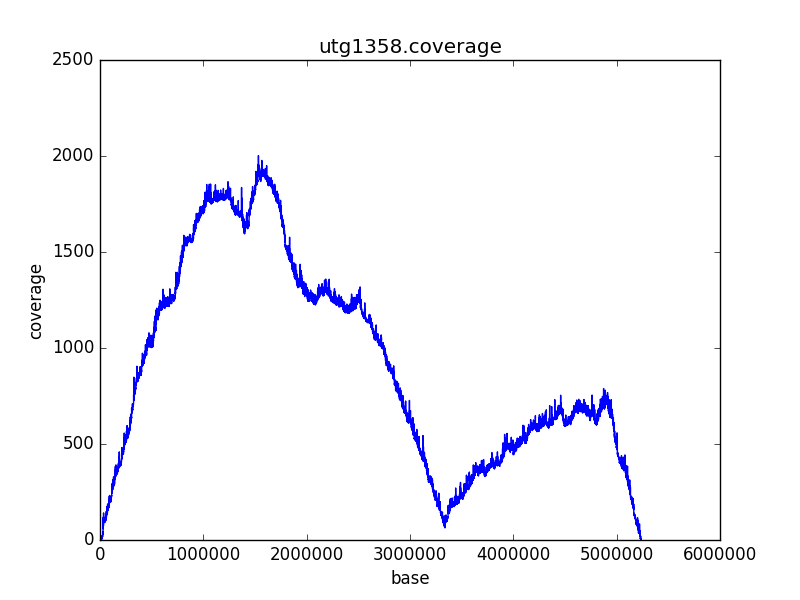


Supplementary Figure S3: Detection of Misassemblies using physical coverage. It can be seen that there is a significant dip in the coverage at about 3.4Mb which indicates a potential misjoin to generate a contig during assembly. This indicates contigs those map to two different chromosomes. Our misassembly detection algorithm finds such dips in the physical coverages and breaks contigs at that position to avoid propagation of errors in the scaffolding process.
